# Effects of different nitrogen fertilization systems on crop yield and nitrogen use efficiency – Results of a field experiment in southern Germany

**DOI:** 10.1016/j.heliyon.2024.e28065

**Published:** 2024-03-14

**Authors:** Martin Mittermayer, Joseph Donauer, Stefan Kimmelmann, Franz-Xaver Maidl, Kurt-Jürgen Hülsbergen

**Affiliations:** Technische Universität München, Chair of Organic Agriculture and Agronomy, Liesel-Beckmann-Straße 2, 85354 Freising, Germany

**Keywords:** Multi-year field experiment, N fertilization, Reduced N supply, German fertilizer application ordinance, Crop rotation

## Abstract

The effects of the German Fertilizer Application Ordinance (GFO) on crop yield, nitrogen use efficiency and economical performance are highly controversial in science and practice in Germany. This study presents the results of a multi-year field experiment conducted at an experimental farm in southern Germany, in which the effects of different fertilizer systems on crop yield, protein concentration and nitrogen balance were analyzed.

At this study site, relatively low N mineralization from the soil N pool was detected. Wheat (*triticum aestivum* L.) and barley (*hordeum vulgare* L.) showed strong yield declines from annual to multi-annual unfertilized plots, for maize (*zea mays* L.), this yield decrease was not observed. The recommendations according to GFO meets the fertilizer requirement at the trial site well. A 20% reduction of fertilization compared to GFO resulted in a 5% yield reduction and a decrease in protein concentration of wheat and barley.

According to the quadratic N response function, the GFO treatment was slightly below the economic optimum nitrogen rate (N_opt_) for wheat, and close to N_opt_ for winter barley on average over the trial years. For maize, a relatively high yield variability has been observed in the trial period so far. Sensor-based fertilization resulted in very high yields with high N use efficiency (up to 85%). This fertilization system can help to reduce nitrogen input and minimize nitrogen surplus.

For wheat and barley, N fertilization and N uptake were well balanced, for maize clearly negative N surpluses were calculated. Despite all the discussion and criticism of GFO, the results of the plot trial show that high yields with high N use efficiency can be achieved with fertilization according to GFO.

## Highlights

• Fertilization according to the German Fertilizer Ordinance leads to high crop yields and high nitrogen use efficiency.
• Sensor-based fertilization can help to reduce nitrogen input and minimize nitrogen surplus.
• The use of realistic target yields is a prerequisite for the correct derivation of the nitrogen fertilizer requirement.
• Reducing nitrogen fertilization by 20% compared to the optimum cause relatively small yield losses (≈5%).
• The regression model has a decisive influence on the determination of the economic optimum nitrogen rate.

## 1 Introduction

Mineral and organic nitrogen (N) fertilization is a key factor in achieving high crop yields and product quality and maintaining soil fertility [[1], [2], [3]]. Crops are unable to take up 100% of the applied N fertilizer, resulting in environmental losses as reactive N [[4], [5], [6], [7]]. Therefore, under practical conditions, determining the optimal N fertilization rate (form, amount, timing) is still challenging, and numerous influencing factors must be considered, for example, crop type, target yield, soil mineral N content (SMN, which is composed of nitrate N and ammonia N), N mineralization, or previous crop. In particular, it is not easy to consider the weather conditions during the growing season and spatially variable soil properties during fertilization [8]. Different systems have been developed to determine the optimal N fertilization rate [9]: (a) simple balance methods that calculate the N fertilizer rate depending on the crop type, target yield, SMN content and other static factors (e.g. the fertilizer requirement calculation according to GFO), (b) methods that additionally consider crop development (biomass and N uptake) at defined growth stages and (c) satellite- and sensor-based site-specific fertilization systems.

The accuracy and reliability of these fertilization systems are controversial in science and agricultural practice. Kage et al. [10] analyzed field trials to evaluate the German Fertilizer Application Ordinance and calculate optimal N fertilization rates for various crops. They compared various mathematical functions (quadratic, quadratic-plateau and linear-plateau) to derive the optimal N fertilization rates. They found that the quadratic plateau function achieved the best fit for their data. As a result of these investigations, the N requirement calculation according to the GFO leads to economic losses compared to the economic optimum. Reducing N fertilization by 20% compared to the recommendations of the GFO, as prescribed in ‘polluted areas’ (areas with high levels of nitrate in the groundwater) [11,12], leads to negative N surpluses in wheat and, in many cases, to grain protein concentrations below the commercial standard. However, Taube [13] came to a contrary conclusion. He stated that the N fertilization recommendation of the GFO is too high. He also criticized the choice of the quadratic-plateau function used by Kage et al. [10] and urged the use of the linear plateau function. The discussion about optimal N fertilization rate is academic as well as it has great relevance for crop production and also a societal dimension because of the multiple environmental impacts and costs [14,15] associated with N use.

### 1.1 Research needs

In Germany, the N balance surplus has been too high for many years at approximately 90 kg ha^−1^ a^−1^, leading to environmentally hazardous N emissions and pollution of drinking water with high NO_3_–N inputs [7]. The German government's sustainability strategy aims to reduce the N surplus from approximately 90 kg ha^−1^ of agricultural land to below 70 kg ha^−1^ by 2030 [16]. Adapted agricultural practices and optimal N fertilization rates also are important for sustainable N use. The German Fertilizer Application Ordinance is mandatory for conventional and organic farms and marks the legally maximum permissible amount of N fertilization in Germany [17,18]. Alternative systems (sensor- or satellite-based) must also not exceed this N fertilization rate. According to the German Fertilizer Application Ordinance [19], fertilizer requirements of winter cereals are calculated before the start of the vegetation in spring, according to yield-dependent reference values, without considering plant growth during the vegetation period or the spatial variability of soil properties of the cropland.

Digital technologies, such as satellite- and sensor-based systems for site-specific N fertilization, consider plant growth and N uptake at the time of fertilization and the spatial variability of yield potential (high and low yield zones within the field) [20,21]. However, the accuracy, efficiency, and application potential of satellite- and sensor-based N fertilization systems are assessed differently [22,23]. This is due to the diversity of methods and their technical implementation, lack of transparency of the underlying fertilization algorithms, or concerns about data security [24,25]. These methods have not yet become widely accepted in agricultural practices in Germany [26].

However, the performance of fertilization systems and the effects of reduced N fertilization (due to restrictions in nitrate N polluted areas) on crop yield, N use efficiency, and N losses have not yet been sufficiently studied, especially the effects at the system level of crop rotations. Therefore, evaluating these fertilizer application systems under differentiated site conditions is necessary. There is no scientific study to date comparing the GFO fertilization strategy (and its modifications, for example, limiting the N demand to 80% of the fertilizer requirement in ‘polluted areas’) with fertilization recommendations using a multispectral sensor-based system.

### 1.2 Subject and aim of this study

The arable farm Roggenstein has been cultivated with cash crops for many decades (>50 years), and only mineral fertilizers have been used (no use of organic fertilizers, no cultivation of legumes). This study evaluated the N fertilization system according to GFO compared to sensor-based N fertilization on a site with high yield potential. In addition, it analyzed how increased and reduced N fertilization rates compared to GFO affects crop yield, protein concentrations, N uptake, N use efficiency, N surplus, and SMN content. The difference between N fertilization according to GFO and optimum N fertilization rate, the crop yield according to GFO, and the optimum crop yield were calculated using N response functions. The effects of the N fertilization systems compared to the unfertilized treatment on yield and N balance were analyzed in three years with different weather conditions (2020, 2021, and 2022) for maize, winter wheat, and winter barley. All three crop types were grown in each experimental year. Compared to previous studies [[27], [28], [29]], the results relate to up-to-date cultivation and fertilization systems. Common varieties were grown, and plant protection and soil cultivation were conducted under practical conditions (Appendix 1-3, exemplary shown for year 2022). Compared with one-year trials, the use of multi-year data is suitable for measuring the possible effects of yield losses or soil N depletion in low fertilized or unfertilized plots. Practical recommendations were made on this basis.

Based on the current scientific knowledge, the hypotheses are.

(1) N fertilization according to GFO is close to the economic optimum.
(2) The reduction in N fertilization (GFO -20%) significantly decreases crop yields and net returns.
(3) Sensor-based N fertilization leads to a significant yield increase compared to GFO, saves mineral N, and simultaneously reduces N balances.
(4) The choice of the right regression function is decisive for the determination of the optimal nitrogen amount and therefore for the evaluation of fertilization systems.

## 2 Materials and methods

### 2.1 Site and weather conditions

The study was carried out at the Roggenstein Research station (48°10′47″ N 11°19′11″ E), 35 km west of Munich (520 m a.s.l.). The farm study areas were characterized by long-term mineral (non-organic) fertilization and crop rotations without forage and grain legumes.

The 30-year mean annual precipitation was 954 mm, and the mean annual air temperature was 8.8 °C (1991–2020) (Table 1). The study years 2020 and 2022 were characterized by a markedly dry and hot summer. Compared with the long-term mean, lower annual precipitation and higher mean air temperatures were measured in 2020 and 2022, respectively (Table 1). 2021 was similar to the long-term air temperature mean, but lower precipitation was measured in the main vegetation phase from April to June. The soils examined at this experimental station were silty loam (medium-quality Cambisols) [30] (Table 2). According to the soil analysis before the start of the plot trial, the nutrient contents were optimal (Table 2).

**Table 1:** Mean temperature (°C) and precipitation (mm), research station Roggenstein.

Year	Jan–Mar	Apr–Jun	Jul–Sep	Oct–Dec	Year
2020					
Temperature x̄	3.0	13.1	17.4	4.8	11.8
Precipitation ∑	185	289	278	134	886
2021					
Temperature x̄	2.0	11.7	16.2	4.5	8.6
Precipitation ∑	155	108	327	46	945
2022					
Temperature x̄	3.3	13.8	17.3	6.5	10.2
Precipitation ∑	57	263	268	206	794
1991–2020					
Temperature x̄	1.2	12.7	16.8	4.3	8.9
Precipitation ∑	172	263	272	202	909

**Table 2:** Characterization of the study field.

Field	Unit	Explanation
Farm		Roggenstein^a^
Coordinates		48°18′53.3″N 11°33′20.4″E
Farming system		Arable Farming
Livestock unit^b^	LU ha^−1^	0
Size of the plot trial (with 3 subtrials)	ha	0.54 ha (3 × 0.18 ha)
Soil		silty loam
Soil type		Cambisol
German soil evaluation^c^		54
pH^d^		5.8
Phosphorus (P_2_O_5_)^d^	mg 100 g^−1^	8.0
Potassium (K_2_O)^d^	mg 100 g^−1^	19
Soil organic nitrogen (SON)^d^	%	0.10
Soil organic carbon (SOC)^d^	%	1.30
Soil mineral nitrogen (SMN) (0–60 cm)^d^	kg ha^−1^	51

a Experimental farm, near Munich.

b Livestock units per hectare.

c 100 = best possible soil quality, Blume et al., 2016.

d Before the beginning of the plot trial 2020 (February 04, 2020).

### 2.2 Experimental design

**Fig. 1:**
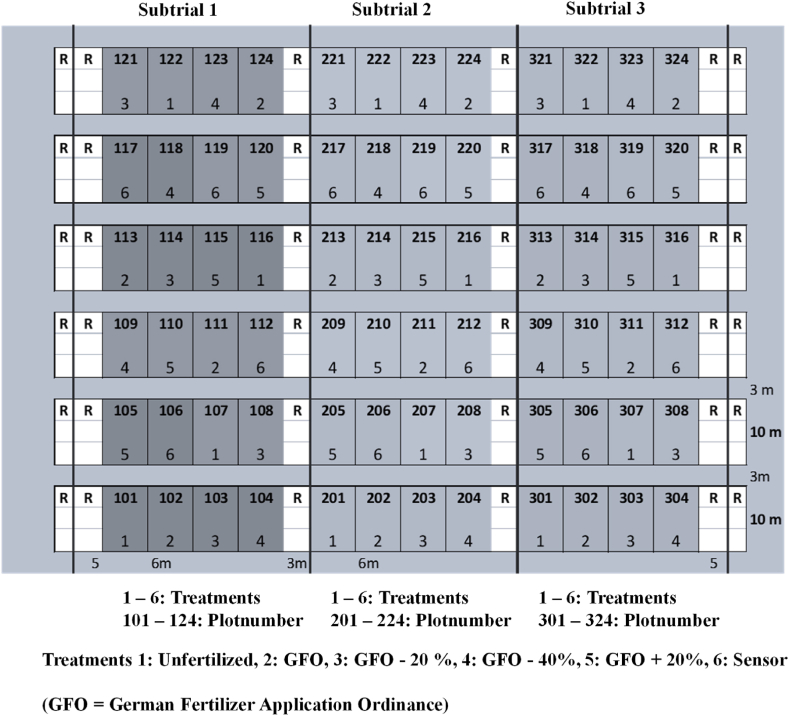
Design of the plot trial, divided in three subtrials. Each subtrial variant (treatment) has four replications. Due to the three subtrials, each crop (maize, wheat, barley) of the three-year crop rotation can be cultivated in each year.

The fertilization experiment is designed as a single-factor trial with 72 plots in three subtrials. Each subtrial had 24 plots with 6 N levels (treatments) with the main factor N fertilization in four replicates (Fig. 1). Other macronutrients and micronutrients were applied according to local farmers practice. The plot size was 60 m^2^ (width: 6 m and length: 10 m). The previous crop was sugar beet. The crop rotation in subtrial 1 was spring wheat (2020), winter barley (2021) and maize (2022); in subtrial 2 spring barley (2020), maize (2021) and winter wheat (2022), and in subtrial 3 maize (2020), winter wheat (2021) and winter barley (2022). Due to the three subtrials, each crop could be grown in each year to investigate the influence of weather conditions. The field experiment is designed as a long-term trial, and the first 3 years of the trial are studied here. The following N fertilization systems were tested.

- N 1 (Unfertilized): No N fertilization
- N 2 (GFO): N fertilization rates according to the GFO
- N 3 (GFO -20%): N fertilization rates lowered by 20 % compared to the GFO
- N 4 (GFO -40%): N fertilization rates lowered by 40 % compared to the GFO
- N 5 (GFO +20%): N fertilization rates increased by 20 % compared to the GFO
- N 6 (Sensor): N fertilization according to the sensor measurement and fertilization algorithm.

In each subtrial, treatments N 1 – N 6 are randomized with four replicates (Fig. 1). The treatments of the experimental plots remain the same throughout the experiment in order to test accumulative effects. Maize, wheat, and barley were analyzed in the trial because (a) these are the most important crops in Germany and the study region, (b) they have a relatively high N fertilization level and (c) sensor-based fertilization systems have already been developed for these crops. The year-specific N fertilization of the crops, differentiated by application and total N fertilizer input, is shown in Table 3.

**Table 3:** N Fertilization and cultivated crops, subtrial 1–3 in the years 2020–2022, separated by different N fertilizer doses (begin of vegetation, BBCH 32, and BBCH 39 for wheat and barley; at sowing and BBCH 15 for maize)^f^.

Year		2020	2021	2022
Subtrial 1	Crop	Spring wheat	Winter barley	Maize
Treatment	Explanation	N Fertilization [kg ha^−1^]	N Fertilization [kg ha^−1^]	N Fertilization [kg ha^−1^]
N 1	Unfertilized	0/0/0 = 0	0/0/0 = 0	0/0 = 0
N 2	GFO^a^	51/60/50 = 161	60/53/60 = 173	137/60 = 197
N 3	GFO - 20%^b^	41/48/40 = 129	43/40/48 = 131	112/48 = 160
N 4	GFO - 40%^b^	31/36/30 = 97	36/36/36 = 108	84/36 = 120
N 5	GFO + 20%^b^	61/72/60 = 193	71/60/72 = 203	165/72 = 237
N 6	Sensor^c^	51/60/50 = 161^d^	60/73/0 = 133	140/60 = 200^d^
Subtrial 2	Crop	Spring barley	Maize	Winter wheat
Treatment	Explanation	N Fertilization [kg ha^−1^]	N Fertilization [kg ha^−1^]	N Fertilization [kg ha^−1^]
N 1	Unfertilized	0/0/0 = 0	0/0 = 0	0/0/0 = 0
N 2	GFO^a^	67/45/0 = 112	124/60 = 184	90/80/66 = 236
N 3	GFO - 20%^b^	54/36/0 = 90	102/48 = 150	72/65/53 = 190
N 4	GFO - 40%^b^	40/27/0 = 67	69/36 = 105	54/55/40 = 149
N 5	GFO + 20%^b^	80/54/0 = 134	137/72 = 209	108/98/79 = 285
N 6	Sensor^c^	67/45/0 = 112^d^	95/60 = 155^e^	60/95/71 = 226
Subtrial 3	Crop	Maize	Winter wheat	Winter barley
Treatment	Explanation	N Fertilization [kg ha^−1^]	N Fertilization [kg ha^−1^]	N Fertilization [kg ha^−1^]
N 1	Unfertilized	0/0 = 0	0/0/0 = 0	0/0/0 = 0
N 2	GFO^a^	167/0 = 167	77/66/66 = 209	60/81/60 = 201
N 3	GFO - 20%^b^	134/0 = 134	62/53/53 = 168	48/70/48 = 166
N 4	GFO - 40%^b^	100/0 = 100	35/40/40 = 115	36/51/36 = 123
N 5	GFO + 20%^b^	200/0 = 200	90/79/79 = 248	72/94/72 = 238
N 6	Sensor^c^	167/0 = 167^d^	60/90/54 = 204	60/97/85 = 242

a Determination of the N fertilizer requirement according to the German Fertilizer Application Ordinance (2020); calculation of N fertilizer requirement see Table 4.

b Modifications of the N fertilizer requirement according to the German Fertilizer Ordinance (2020).

c Determination of the N fertilizer requirement based on multispectral sensor data and algorithm according to Maidl (2011).

d No sensor fertilization for maize, summer barley, and summer wheat (fertilization was carried out according to the German Fertilizer Application Ordinance (2020)).

e The N fertilization demand was calculated: the expected N uptake at harvest – 20 %, and consideration of soil mineral nitrogen content at the beginning of vegetation.

f HESS et al. (1997): Use of the extended BBCH scale.

Spring barley and spring wheat were grown in the first year of the trial (2020), as the trial was set up in spring. Winter wheat and barley have been cultivated since 2021. Soil samples were collected at different dates (beginning of vegetation, after harvest, and autumn) in 2020 and 2021 in each treatment and from 2022 in each plot to analyze the mineral N content.

The N fertilizer requirement (FR_fo_) according to the German Fertilizer Application Ordinance [19] is calculated based on the N requirement at the standard yield level (NR_sy_) (for wheat 8 t ha^−1^) plus the yield deviation between the expected and standard yield levels (YD) multiplied by a crop-specific coefficient (a), which compensates for the additional or lower N demand according to the yield deviation (Table 4, equation (1)). The soil mineral N content at the start of the vegetation phase in spring (SMN_vs_), the N delivery (N mineralization) from soil humus (D_hu_), the N delivery from organic fertilization in the previous year (D_of_), and the N delivery from previous crop residues (D_pc_) are considered and reduce the N fertilizer amount. D_hu_ and D_pc_ as well as the proportion of what is mineralized from organic fertilizers (concerns D_of_) are static coefficients that can be taken from tables in the German Fertilizer Ordinance (GFO 2020). In years with unusually poor crop development of winter cereals during autumn and winter, a nitrogen surcharge (S_cd_) of 10 kg ha^−1^ is considered.

(1) $${\text{FR}}_{\text{fo}}={\text{NR}}_{\text{sy}}+\mathrm{YD}*a-\left(\;{\text{SMN}}_{\text{vs}}+D_{\text{hu}}+\sum D_{\text{of}}+\sum D_{\text{pc}}\;\right)+S_{\text{cd}}$$

In N 6 (sensor) treatment, the 1st N application in barley and wheat, which takes place at vegetation start, was set to the same N input as for treatment N 2 (GFO) in 2020 and to 60 kg ha^−1^ in the following years. Therefore, the 1st N application is always independent of reflection measurements, because at this time the still low biomass of the plant stand does not yet allow reliable sensor measurements. For the 2nd and 3rd N application at BBCH 32 and BBCH 39 respectively, the fertilizer requirement is determined by using a handheld multispectral sensor (TEC5 2010), the calculation of the vegetation index REIP (Red Edge Inflection Point), and the application of an N fertilization algorithm according to Maidl [31,32] (equations (2) and (3)).

**Table 4:** Parameters of the fertilizer requirement calculation according to the German Fertilizer Application Ordinance (equation (1)).

Crop	Unit	Wheat 2020	Wheat 2021	Wheat 2022	Barley 2020	Barley 2021	Barley 2022	Maize 2020	Maize 2021	Maize 2022
${FR}_{sm}$	kg ha^−1^	161	209	236/239/242/240^1^	112	173	201/204/	167	184	195/202/
							203/205^1^			196/195^1^
${NR}_{sy}$	kg ha^−1^	220	230	230	140	180	180	200	200	200
YD	t ha^−1^	1.4	2	2	4.5	3	3	20	10	10
Y_e_	t ha^−1^	8.4	10.0	10.0	9.5	10.0	10.0	65.0	55.0	55.0
S_y_	t ha^−1^	7.0	8.0	8.0	5.0	7.0	7.0	45.0	45.0	45.0
a	kg t^−1^	10	10	10	10	10	10	2	2	2
${SMN}_{vs}$	kg ha^−1^	63	41	24/21/18/20^1^	63	37	9/6/7/5^1^	63	36	25/18/24/25^1^
$D_{hu}$	kg ha^−1^	0	0	0	0	0	0	0	0	0
$D_{of}$	kg ha^−1^	0	0	0	0	0	0	0	0	0
$D_{pc}$	kg ha^−1^	10	0	0	10	0	0	10	0	0
$S_{cd}$	kg ha^−1^	0	0	10	0	0	0	0	0	0

FR_sm_ (Fertilizer requirement), NR_sy_ (Nitrogen requirement at standard yield level), YD (Yield deviation between expected and standard yield), Y_e_ (Fresh matter yield expectation)), S_y_ (Standard yield), a (coefficient), SMN_vs_ (Soil mineral N at vegetation start), D_hu_ (Nitrogen delivery from soil humus), D_of_ (Nitrogen delivery from organic fertilization in the previous years), D_pc_ (Nitrogen delivery from previous crop residues), $S_{cd}$ (Surcharge for poor crop development in spring for cereals).

From the year 2022 on, in each plot soil mineral nitrogen content was taken separately. This resulted in four different fertilization rates.

Spring barley, spring wheat, and maize were not fertilized according to the sensor and algorithm, as no algorithms for N fertilization are (yet) available. Therefore, in treatment N 6, spring wheat and barley were fertilized according to the GFO. In 2020 and 2022 maize was also fertilized according to the GFO, and in 2021, the N fertilizer requirement was calculated as the expected N uptake −20%.

The calculation of the N fertilizer requirement for winter wheat and winter barley according to the sensor-based method (FR_s_) considers various parameters (equation (2)). The calculations were based on N uptake functions derived from field experiments. They describe crop-specific how much N a plant stand should have taken up at a defined growth stage to achieve the target yield. To determine the N fertilizer requirement, the differences between the target value of N uptake at the next fertilization date (NU_tnf_) and the target value of N uptake at the current fertilization date (NU_tcf_), as well as the target value of N uptake at the current fertilization date (NU_tcf_) and the actual N uptake at the current fertilization date (NU_c_) are calculated. The DIMA factor in equation (2) corresponds to the expected crop and site-specific nitrogen efficiency, theoretically can assume values between 0 and 1, but usually is around 0.7 for winter wheat and winter barley. The DIMA factor was determined experimentally. It takes into account the duration of the mineral fertilization effect, the immobilization rate of the fertilizer N, the mineralization of soil N, and the utilization factor of the fertilizer [32].

(2) $${\text{FR}}_{s}=\left({\mathrm{NU}}_{\text{tnf}}-{\text{NU}}_{\text{tcf}}+\left({\text{NU}}_{\text{tcf}}-{\text{NU}}_{c}\right)\right)*\;\mathrm{DIMA}$$

The REIP vegetation index correlates strongly to N uptake. Using a quadratic function, the N uptake was estimated based on the REIP vegetation index at reflection at 670 nm (A), 700 nm (B), 740 nm (C) and 800 nm (D).

(3) REIP=700+40*((A+D)/2−B)/(C−B)

### 2.3 Crop yield, protein concentrations, and N uptake

The yield data for each plot were determined using a plot combine harvester [33]. The inner 15 m^2^ of the plots were harvested to prevent the influence of neighboring plots and edge effects.

Laboratory analyses were performed to determine the grain dry matter content after drying at 60 °C. Fresh matter (FM) yields (in t ha^−1^) were calculated based on 86% dry matter (DM) content for wheat and barley and based on 32% DM content for silage maize. Grain N content was determined using a Vario MAX cube C/N Analyzer [34]. The N uptake was determined by multiplying the grain yield by the N content from laboratory analyses.

Byproducts (straw) were not harvested or considered as N output.

### 2.4 Nitrogen balancing

The N surplus (kg ha^−1^) was determined by a simple N balancing method:

(4) $$N_{sur}=N_{in}-N_{out}$$

N surplus (N_sur_) describes the potential loss of reactive N compounds (NH_3_, N_2_O, and NO_3_^−^).

The N use efficiency (NUE) (%) was determined using the difference method:

(5) $$\mathrm{NUE}=\left(N_{out\_ft}-N_{out\_uft}\right)\;/\;N_{\text{in}}$$

The N input (N_in_) was the amount of mineral N fertilizer that was applied to each plot (Table 3). The N output (N_out_) was the grain N uptake in the fertilized (N_out_ft_) and unfertilized treatment (N_out_uft_), respectively.

### 2.5 Soil sampling for determination of soil mineral nitrogen content

Soil samples were collected at two depths (0–30 and 30–60 cm) to determine SMN content (nitrate N and ammonia N content). Usually, soil depth 60–90 cm should also be taken into account but at the study site rooting depth ends at 60 cm. Below that there is only gravel. The samples were collected for each treatment at the beginning of vegetation 2021 and for each plot after harvest 2021, in autumn 2021, at the beginning of vegetation 2022, after harvest 2022, and in autumn 2022.

### 2.6 Statistical analyses

The statistical processing and data analysis were conducted with “R” [35]. The packages used in “R” are “agricolae” and “stats”. For the analysis of variance, the data were checked for a normal distribution of the factors to be examined. In addition, the Shapiro-Wilk test was performed using the “shapiro.test” function to test for normal distribution. Subsequently, the analysis of variance (ANOVA) was calculated using the function “aov.” The N fertilizer factor was checked for dependent variables such as yield, protein concentrations, N uptake, N surplus, N use efficiency, and SMN content. If the F-test of the ANOVA showed significance, a further test procedure was used to determine which treatments differed significantly. For this purpose, the Student-Newman-Keuls test was applied as a post-hoc test. A significant difference at the p < 0.05 level was used to separate means attached with different letters (a-e).

To evaluate the fertilizer systems, N response functions were fitted to predict crop fresh matter yields (Y_fm_) as a function of N fertilization (N_fert_). As the choice of the prediction model may have an influence on the predicted N fertilizer amount reaching the maximum and economic optimum yield, three different models were parameterized for each crop in each year. The first is the quadratic function, which was calculated according to equation (6) using the lm-procedur.

(6) $$Y_{fm}=a*{N_{\text{fert}}}^{2}+b*N_{\mathrm{fert}\;}+c$$

The second is the quadratic-plateau function (equation (7)), and the third is the linear-plateau function (equation (8)). These functions were calculated using the nls procedure.

(7) $$Y_{fm}=\left\{\begin{array}{c} {b*\left(N_{\text{fert}}-c\right)}^{2}+a\\ a \end{array}\right\}\;\begin{array}{c} N_{\text{fert}}\leq c\\ N_{\text{fert}}>c \end{array}$$

(8) $$Y_{fm}=\left\{\begin{array}{c} b*\left(N_{\text{fert}}-c\right)+a\\ a \end{array}\right\}\;\begin{array}{c} N_{\text{fert}}\leq c\\ N_{\text{fert}}>c \end{array}$$

For the derivation of the economic optimum N level, a price of 1 € kg^−1^ N for fertilizers (P_fert_), a product price (P_prod_) of 200 € t^−1^ FM at 86% for wheat, 170 € t^−1^ FM at 86% for barley, and 28,80 € t^−1^ FM for silage maize were assumed. A difference in the product price for wheat resulting from different crude protein concentrations or baking qualities was not considered. Additional transportation costs due to higher fertilizer and harvest amounts were neglected, making N fertilizer costs the only variable. Appendix 8 examplary shows revenue response curves for barley 2020.

The economic optimum N level is defined as the nitrogen fertilizer amount, where the marginal costs for an extra unit N fertilizer equals the marginal revenue gained by applying this extra unit of N fertilizer (equation (9)). In Appendix 8, the maximum of the functions corresponds to the economic optimum N fertilizer level.

For the linear plateau function according to equation (8) mathematically there are just two values for marginal revenue possible – either, when N_fert_ ≤ c, the calculated value for b, which is larger than 0 or 0, when N_fert_ > c. In consequence, the economic optimum N level is 0, when b ≤ P_fert_ or b, when b > P_fert_.

(9) $$\Delta N_{\text{fert}}*P_{\text{fert}}={\Delta Y}_{\text{fm}}*P_{\text{prod}}$$

For the relationship between nitrogen fertilization and yield, there is ample evidence that shows a functional relationship (e.g. quadratic, quadratic-plateau and linear-plateau) between the two variables [36,37]. For nitrogen balances, on the other hand, there are hardly any sources that describe the functional relationship between N fertilization and N balance. A decreasing increase in N uptake with increasing fertilization levels rules out a linear relationship between N fertilization and N balance. The following three functions, equation (10), equation (11), and equation (12) were therefore parameterized for the estimation of the N surplus in the range of the tested N levels.

(10) $$N_{sur}=a*{N_{\text{fert}}}^{2}+b*N_{\mathrm{fert}\;}+c$$

(11) $$N_{sur}=\left\{\begin{array}{c} {b*\left(N_{\text{fert}}-c\right)}^{2}+a\\ a \end{array}\right\}\;\begin{array}{c} N_{\text{fert}}\geq c\\ N_{\text{fert}}<c \end{array}$$

(12) $$N_{sur}=\left\{\begin{array}{c} b*\left(N_{\text{fert}}-c\right)+a\\ a \end{array}\right\}\;\begin{array}{c} N_{\text{fert}}\geq c\\ N_{\text{fert}}<c \end{array}$$

These functions were used to calculate the N surplus at the calculated economically optimal fertilizer level (N_opt_) and the fertilizer rate that produces the maximum yield (N_max_).

#### 2.6.1 Regression coefficients

The fitted coefficients of equations (6), (7), (8) for yields and equation (10), (11), (12) for N surplus can be found in Appendices 4 and 5, respectively. Table 8 shows the associated assessment values mean absolute error (MAE), root mean square error (RMSE), and the coefficient of determination (R^2^) as well as the model characteristics N_max_ ((minimum) N fertilizer amount to reach maximum yield), Y_max_ (maximum yield), N_opt_ (N fertilizer amount to get the maximum economic output), and Y_opt_ (yield at N_opt_) for the yield data.

## 3 Results

### 3.1 Wheat

#### 3.1.1 Spring wheat; year 2020; subtrial 1

In 2020, wheat grain yield in treatment N 1 (unfertilized) was 5.0 t ha^−1^ (Table 5). All fertilized treatments exceeded the target yield of 8.4 t ha^−1^. The highest yield was achieved in treatment N 5 (GFO +20%) with 10.8 t ha^−1^, although this was not statistically different from the yield of treatment N 2 (GFO) (10.3 t ha^−1^) and N 6 (Sensor) (10.4 t ha^−1^). N 3 (GFO -20%) did not result in a significantly lower yield compared to N 2 (GFO); however, N 4 (GFO -40%) reduced the yield significantly. Increasing N fertilizer application significantly increased protein concentrations from 8.7% (N 1, unfertilized) to 13.6% in the highest fertilized treatment N 5 (GFO +20%). N uptake was 60 kg ha^−1^ in treatment N 1 (unfertilized), and the highest N uptake was measured in treatment N 5 (GFO +20%) with 199 kg ha^−1^, with N fertilization of 193 kg ha^−1^. The N use efficiency varied only slightly. The highest N use efficiency was achieved in treatments N 3 (GFO -20%) (75%) and N 6 (Sensor) (75%), and these differed statistically only from treatment N 4 (GFO -40%), which showed yield losses owing to low N fertilization. Under experimental conditions, only negative N balances were determined; increasing N fertilization achieved an almost balanced N surplus in treatment N 5 (GFO +20%).

**Table 5:** Yields and N balances of spring wheat in the year 2020 and of winter wheat in the years 2021 and 2022.

Treatment Unit	Explanation	FM yield [t ha^−1^]	Protein [%]	N uptake [kg ha^−1^]	N surplus [kg ha^−1^]	NUE [%]
Spring wheat; year 2020; subtrial 1						
N 1	Unfertilized	5.0^a^	8.7^a^	60^a^	−60^a^	
N 2	GFO^a^	10.3^c^	12.4^cd^	176^d^	−15^b^	72^b^
N 3	GFO - 20%^b^	9.7^c^	11.8^c^	158^c^	−29^b^	75^b^
N 4	GFO - 40%^b^	8.9^b^	10.4^b^	127^b^	−30^b^	69^a^
N 5	GFO + 20%^b^	10.8^c^	13.6^e^	199^e^	-6^c^	72^b^
N 6	Sensor^c^	10.4^c^	12.9^de^	181^d^	−20^c^	75^b^
Winter wheat; year 2021; subtrial 3						
N 1	Unfertilized	2.0^a^	8.3^a^	26^a^	−26^a^	
N 2	GFO^a^	10.7^d^	11.5^d^	187^d^	21^c^	77^ab^
N 3	GFO - 20%^b^	10.0^c^	10.5^c^	160^c^	8^b^	80^b^
N 4	GFO - 40%^b^	7.9^b^	9.3^b^	112^b^	3^b^	75^a^
N 5	GFO + 20%^b^	11.2^d^	12.5^e^	213^e^	34^d^	75^a^
N 6	Sensor^d^	11.1^d^	11.5^d^	194^d^	16^c^	82^b^
Winter wheat; year 2022; subtrial 2						
N 1	Unfertilized	2.3^a^	7.8^a^	28^a^	−28^a^	
N 2	GFO^a^	11.4^e^	11.7^d^	201^d^	37^d^	73^b^
N 3	GFO - 20%^b^	10.6^c^	10.5^c^	168^c^	24^c^	74^b^
N 4	GFO - 40%^b^	9.3^b^	9.2^a^	131^b^	15^b^	69^a^
N 5	GFO + 20%^b^	11.4^e^	12.8^e^	220^e^	66^e^	67^a^
N 6	Sensor^d^	10.9^d^	11.8^d^	196^d^	35^d^	74^b^

a Determination of the N fertilizer requirement according to the German Fertilizer Ordinance (2020).

b Modifications of the N fertilizer requirement according to the German Fertilizer Ordinance (2020).

c No sensor fertilization for summer barley (fertilization was carried out according to the German Fertilizer Ordinance (2020)).

d Determination of the N fertilizer requirement based on multispectral sensor data and algorithm according to Maidl (2011).

**Fig. 2:**
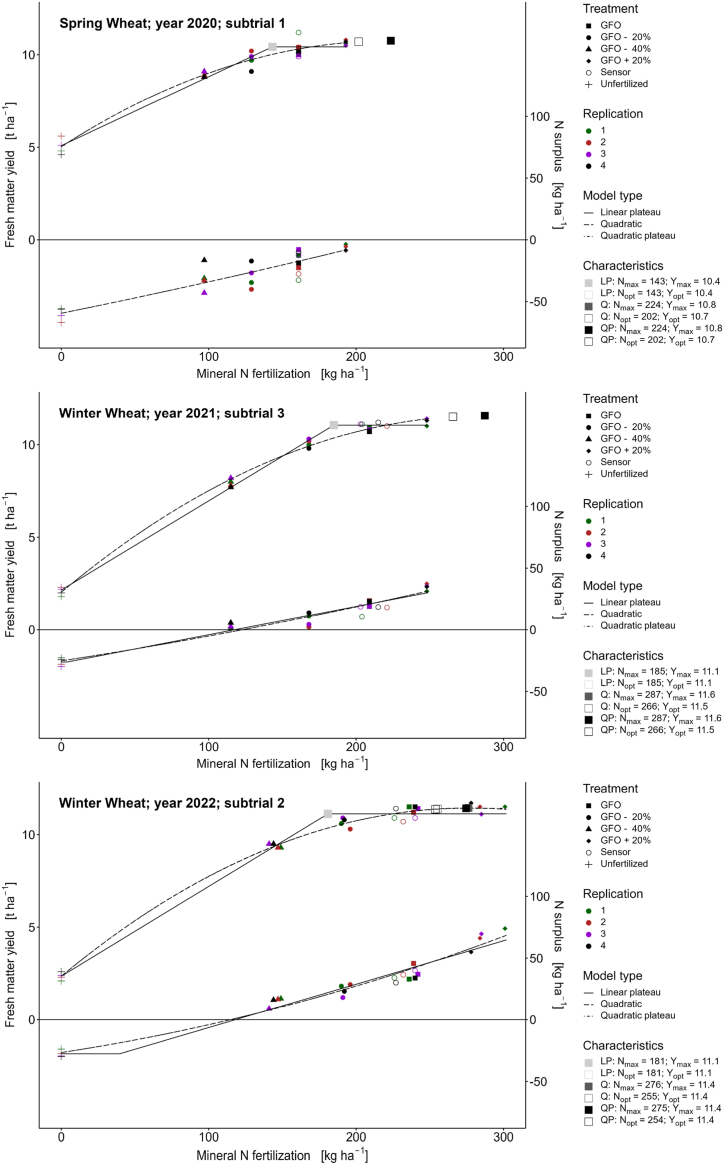
Relationships between N fertilization, yield, and nitrogen surplus for wheat. Yield is given in fresh matter with a dry matter content of 86%.

The calculated economic optimum yield was achieved at a fertilizer N input of 143 kg ha^−1^ (LP model) and 202 kg ha^−1^ (Q and QP model), respectively (Fig. 2, subtrial 1). Thus, according to the Q and QP models, the economic optimum was not reached even in treatment N 5, with 20% higher N fertilization than treatment N 2 (GFO). The yield at the economically optimum N fertilization rate exceeded the target yield by more than 2 t ha^−1^ (Table 8).

#### 3.1.2 Winter wheat; year 2021; subtrial 3

In the second year of the trial, an extremely low yield of 2.0 t ha^−1^ was achieved in treatment N 1 (unfertilized) (Table 4). All fertilized treatments exceeded the target yield of 10.0 t ha^−1^, except N 4 (GFO -40%). Treatment N 2 (GFO) did not achieve the highest yield, but treatment N 5 (GFO +20%) (11.2 t ha^−1^) and treatment N 6 (Sensor) (11.1 t ha^−1^) did. In treatment N 6 (Sensor), less N was applied than in treatment N 2 (GFO), but the N distribution was different (less N in the first application, more in the second application in N 6). The treatments with reduced N fertilization (N 3 and N 4) resulted in significantly lower yields. The increase in fertilizer rate in N 5 (GFO +20%) (248 kg ha^−1^) compared to N 6 (Sensor) (204 kg ha^−1^) did not increase yield.

The protein concentrations was strongly differentiated depending on fertilization, from 8.3% in the unfertilized treatment (N 1) to 12.5% in the highly fertilized treatment (N 5). The N uptake of the unfertilized treatment was extremely low (26 kg ha^−1^). The highest N uptake was observed in treatment N 5 (GFO +20%) (213 kg ha^−1^).

The N use efficiency was relatively high, ranging from 75% in N 4 (GFO -40%) and N 5 (GFO +20%) to 82% in N 6 (Sensor), and showed statistically significant differences. Due to the relatively high yield and N uptake, relatively low N surplus (3–34 kg ha^−1^) were obtained in the fertilized treatments.

The slope of the N response function (Fig. 2, subtrial 3) was steep, which can also be attributed to the low yields in treatment N 1 (unfertilized). The economic optimum, according to the Q and QP models (N_opt_ = 266 kg ha^−1^, Y_opt_ = 11.5 t ha^−1^), was not reached with any of the fertilizer treatments. On the contrary, according to the LP model, the economic optimum yield was reached with a fertilizer N quantity of 185 kg ha^−1^ with 11.1 t ha^−1^ grain yield (Table 8).

#### 3.1.3 Winter wheat; year 2022; subtrial 2

In 2022, to compensate for poor crop development compared to normal years, a 10 kg ha^−1^ N supplement was applied for all fertilizer treatments (except treatment N 6, sensor) before adjusting by ± 20% or −40% (Table 5). In the third trial year, very low yields were achieved in treatment N 1 (unfertilized) with 2.3 t ha^−1^. All fertilized treatments exceeded the target yield of 10 t ha^−1^, except N 4 (GFO -40%). The highest yields were achieved in N 2 (GFO) and N 5 (GFO +20%) with 11.4 t ha^−1^. Lower N applications in N 3 (GFO -20%) and N 4 (GFO -40%) significantly reduced yield and led to reduced protein concentrations. N 6 (Sensor) did not result in maximum yield; the difference to the maximum yield was 0.5 t ha^−1^. The additional N application of 49 kg ha^−1^ in treatment N 5 (GFO +20%) did not increase yield compared to N 2 (GFO) but resulted in the statistically highest protein concentration (12.8%) and the highest N uptake (220 kg ha^−1^). However, N fertilization of 285 kg ha^−1^ in N 5 (GFO +20%) resulted in the lowest N use efficiency (67%). The highest N use efficiency was achieved in N 3 (GFO -20%) and N 6 (Sensor), with 74% each. The highest N surplus (66 kg ha^−1^) was reached in the highest fertilizer treatment (N 5, GFO +20%) and showed statistically significant differences from all treatments. In the treatments N 2 (GFO), N 3 (GFO -20%), N 4 (GFO -40%), and N 6 (Sensor), relatively balanced N surplus (15 kg ha^−1^ to 37 kg ha^−1^) were determined.

In 2022, according to the LP, QP, and Q models, the economic optimum N rate amounted to 181 kg ha^−1^, 254 kg ha^−1^, and 255 kg ha^−1^ (Table 8). Fertilization according to GFO (236 kg ha^−1^) was 55 kg ha^−1^ above the theoretical optimum (LP model) and 18 resp. 19 kg ha^−1^ below the theoretical optimum (QP and Q model).

The calculated N surplus at the economic optimum remained below the critical value of 50 kg N ha^−1^, regardless of which model type was considered (Fig. 2, subtrial 2, and Appendix 6).

### 3.2 Barley

#### 3.2.1 Spring barley; year 2020; subtrial 2

In 2020, a barley grain yield of 6.4 t ha^−1^ was measured in treatment N 1 (unfertilized). N 2 (GFO), N 3 (GFO -20%), and N 6 (Sensor) exceeded the target yield of 9.5 t ha^−1^. An additional N supply (GFO +20%) in treatment N 5 significantly reduced the yield compared to treatment N 2 (GFO), indicating overfertilization. With increasing fertilization, protein concentration increased significantly from 7.8% in N 1 (unfertilized) to 12.6% in the highest fertilized treatment, N 5 (GFO +20%). In treatments N 2, N 5, and N 6, almost the same N uptake was attained (153–156 kg ha^−1^); in treatment N 1, the N uptake was 68 kg ha^−1^.

**Fig. 3:**
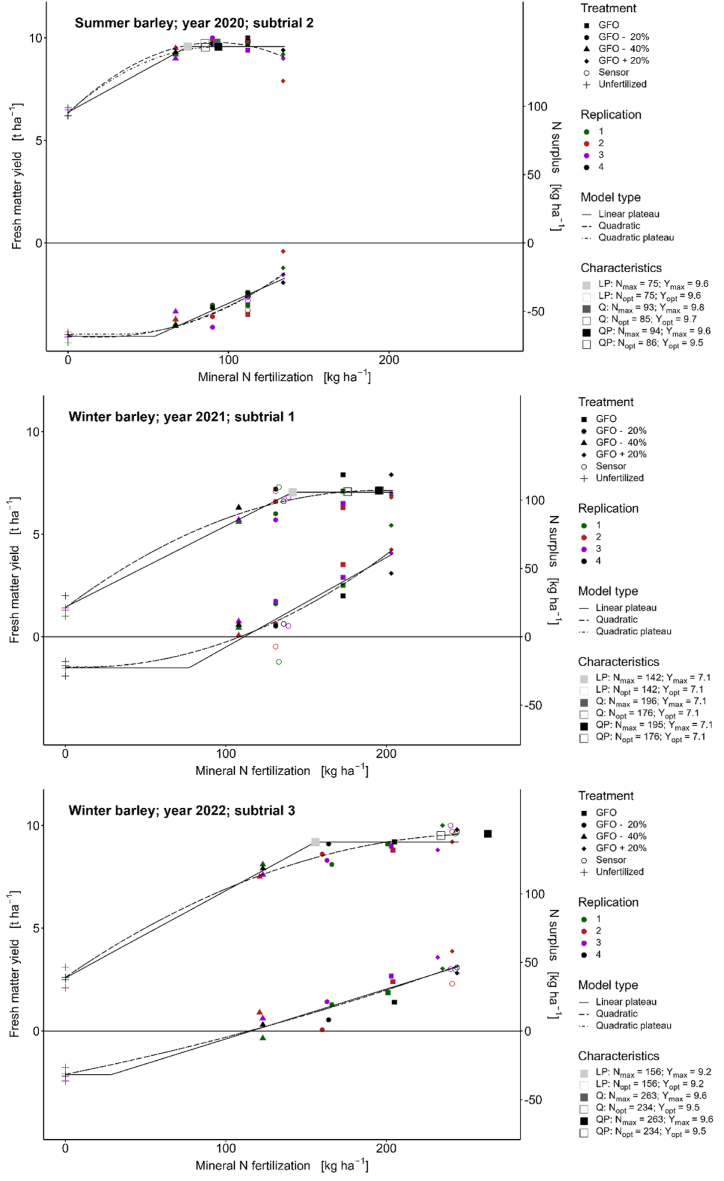
Relationships between N fertilization, yield, and nitrogen surplus for barley. Yield is given in fresh matter with a dry matter content of 86%.

Owing to low N fertilization and high yields, relatively high N use efficiency (82%) and strongly negative N surplus (−56 to −52 kg ha^−1^) were observed in the treatments N 3 (GFO -20%) and N 4 (GFO -40%). Negative N balances were found (−19 kg ha^−1^) even in the highest fertilized treatment N 5 (GFO +20%). Owing to the relatively high yield of the unfertilized treatment N 1 and the flat slope of the N response function (Fig. 3), the optimum yield (Y_opt_ 9.5 to 9.8 t ha^−1^) was already achieved with N applications of 75 kg ha^−1^ (LP model), 85 kg ha^−1^ (Q model), and 86 kg ha^−1^ (QP model) (Fig. 3). The treatment N 2 (GFO) evidently exceeded the optimal N application with an N supply of 112 kg ha^−1^.

#### 3.2.2 Winter barley; year 2021; subtrial 1

In 2021, the target yield of 10.0 t ha^−1^ was not reached for winter barley in any treatment. The winter barley yield was relatively low in the trial that year. In treatment N 1 (unfertilized), an extremely low yield of 1.4 t ha^−1^ was recorded (Table 6). The highest yields were in treatments N 2 (GFO), N 5 (GFO +20%), and N 6 (sensor). However, 40 kg ha^−1^ less mineral N was applied in the sensor treatment than in the GFO treatment, and 70 kg ha^−1^ mineral N was saved compared with treatment N 5 (GFO +20%).

**Table 6:** Yields and N balances of spring barley in the year 2020 and of winter barley in the years 2021 and 2022.

Treatment Unit	Explanation	FM yield [t ha^−1^]	Protein [%]	N uptake [kg ha^−1^]	N surplus [kg ha^−1^]	NUE [%]
Spring barley; year 2020; subtrial 2						
N 1	Unfertilized	6.4^a^	7.8^a^	68^a^	−68^a^	
N 2	GFO^a^	9.8^c^	11.6^d^	155^c^	−43^c^	77^b^
N 3	GFO - 20%^b^	9.9^c^	10.5^c^	142^c^	−52^bc^	82^c^
N 4	GFO - 40%^b^	9.2^b^	9.7^b^	123^b^	−56^b^	82^c^
N 5	GFO + 20%^b^	8.9^b^	12.6^e^	153^c^	−19^d^	63^a^
N 6	Sensor^c^	9.8^c^	11.9^e^	156^c^	−41^c^	76^b^
Winter barley; year 2021; subtrial 1						
N 1	Unfertilized	1.4^a^	11.9^a^	22^a^	−22^a^	
N 2	GFO^a^	6.9^c^	13.8^b^	132^b^	40^c^	63^b^
N 3	GFO - 20%^b^	6.3^bc^	13.0^ab^	114^b^	16^b^	70^c^
N 4	GFO - 40%^b^	5.8^b^	12.6^ab^	101^b^	7^ab^	73^c^
N 5	GFO + 20%^b^	7.1^c^	14.3^b^	139^b^	63^d^	57^a^
N 6	Sensor^d^	6.9^c^	14.2^b^	136^b^	-2^b^	85^d^
Winter barley; year 2022; subtrial 3						
N 1	Unfertilized	2.5^a^	8.9^a^	32^a^	−32^a^	
N 2	GFO^a^	9.0^cd^	13.8^d^	172^d^	31^c^	70^b^
N 3	GFO - 20%^b^	8.5^c^	12.9^c^	150^c^	12^b^	71^b^
N 4	GFO - 40%^b^	7.7^b^	10.9^b^	117^b^	5^b^	69^b^
N 5	GFO + 20%^b^	9.5^d^	14.5^de^	188^e^	49^d^	64^a^
N 6	Sensor^d^	9.7^d^	14.8^e^	199^e^	42^d^	71^b^

a Determination of the N fertilizer requirement according to the German Fertilizer Ordinance (2020).

b Modifications of the N fertilizer requirement according to the German Fertilizer Ordinance (2020).

c No sensor fertilization for summer barley (fertilization was carried out according to the German Fertilizer Ordinance (2020)).

d Determination of the N fertilizer requirement based on multispectral sensor data and algorithm according to Maidl (2011).

The protein concentration in N 1 (unfertilized) was 11.9% and increased by N fertilization up to 14.3% in N 5 (GFO +20%). Grain N uptake increased with additional N fertilizer applications up to a maximum of 139 kg ha^−1^ in N 5 (GFO +20%). N use efficiency varied widely this year, ranging from 57% (N 5, GFO +20%) to 85% (N 6, sensor). The calculated N balances varied from −22 kg ha^−1^ in N 1 (unfertilized) to 63 kg ha^−1^ in the highest fertilizer treatment (N 5, GFO +20%).

Fertilization according to N 2 (GFO) (173 kg ha^−1^) nearly met the economically optimal amount of fertilizer according to the Q model (176 kg ha^−1^) and QP model (176 kg ha^−1^) but apparently exceeded the optimal N application according to the LP model (142 kg ha^−1^) (Table 8, Fig. 3).

#### 3.2.3 Winter barley; year 2022; subtrial 3

In 2022, the target yield of winter barley (10.0 t ha^−1^) was not achieved in any fertilization treatment. N 6 (Sensor) (9.7 t ha^−1^) and N 5 (GFO +20%) (9.5 t ha^−1^) achieved the maximum yield (Table 6). Both treatments had higher N inputs than N 2 (GFO). The fertilization according to N 2 (GFO) resulted in a yield of 9.0 t ha^−1^, which was not significantly different from the yield of the treatments N 5 (GFO +20%) and N 6 (Sensor). The highest protein concentration of 14.8% and N uptake (199 kg ha^−1^) were obtained in the treatment in N 6 (Sensor). The lowest N use efficiency (64%) and the highest N surplus (49 kg ha^−1^) were found in treatment N 5 (GFO +20%).

The economic optima according to the LP model were N_opt_ = 156 kg ha^−1^ and Y_opt_ = 9.2 t ha^−1^; according to the Q and QP model N_opt_ = 234 kg ha^−1^ and Y_opt_ = 9.5 t ha^−1^ (Table 8, Fig. 3). Thus, fertilization according to GFO (treatment N 2) was below, and treatment N 6 (sensor) was slightly above the economic optimum in 2022. Fertilization according to the optimum of the Q or QP model did not exceed the critical N surplus of 50 kg ha^−1^ (Fig. 3, Appendix 6).

### 3.3 Maize

#### 3.3.1 Maize; year 2020; subtrial 3

In 2020, only one N application was applied to maize (Table 3). The target yield of 65.0 t ha^−1^ (Table 4) was not reached in any treatment. In the trial, generally low yields were recorded in 2020, which can be attributed to specific weather conditions. In treatment N 1 (unfertilized), yield was 30.7 t ha^−1^ (Table 7). The yields (37.8–41.8 t ha^−1^) of the fertilized treatments were not statistically different. Relatively high N uptake was measured in N 1 (unfertilized) at 111 kg ha^−1^. The highest N uptake was achieved in N 5 (GFO +20%) and N 6 (Sensor) with 185 kg ha^−1^, respectively, 186 kg ha^−1^. N use efficiency was relatively low, ranging from 36% in N 2 (GFO) to 44% in N 3 (GFO -20%) and N 6 (Sensor). Only in treatment N 5 (GFO +20%) positive N balances (14 kg ha^−1^) were determined.

**Table 7:** Yields and N balances of maize in the years 2020–2022.

Treatment Unit	Explanation	FM yield [t ha^−1^]	N uptake [kg ha^−1^]	N surplus [kg ha^−1^]	NUE [%]
Maize; year 2020; subtrial 3					
N 1	Unfertilized	30.7^a^	111^a^	−111^a^	
N 2	GFO^a^	40.6^b^	172^bc^	-5^cd^	36^a^
N 3	GFO - 20%^b^	39.3^b^	171^bc^	−37^b^	44^b^
N 4	GFO - 40%^b^	37.8^b^	147^b^	−47^b^	36^a^
N 5	GFO + 20%^b^	41.5^b^	185^c^	14^d^	37^a^
N 6	Sensor^c^	41.8^b^	186^c^	−19^bc^	44^b^
Maize; year 2021; subtrial 2					
N 1	Unfertilized	36.0^a^	100^a^	−100^a^	
N 2	GFO^a^	61.7^c^	253^c^	−69^bc^	83^c^
N 3	GFO - 20%^b^	54.5^bc^	203^b^	−53^c^	68^a^
N 4	GFO - 40%^b^	50.0^b^	190^b^	−85^ab^	85^c^
N 5	GFO + 20%^b^	60.3^c^	261^c^	−52^c^	77^b^
N 6	Sensor^c^	56.2^bc^	214^b^	−59^c^	74^b^
Maize; year 2022, subtrial 1					
N 1	Unfertilized	42.7^a^	115^a^	−115^a^	
N 2	GFO^a^	52.5^b^	249^b^	−53^ab^	68^b^
N 3	GFO - 20%^b^	50.0^b^	202^b^	−43^ab^	54^a^
N 4	GFO - 40%^b^	49.7^b^	194^b^	−74^ab^	66^b^
N 5	GFO + 20%^b^	52.1^b^	255^b^	−18^b^	59^a^
N 6	Sensor^c^	53.7^b^	253^b^	−52^ab^	68^b^

a Determination of the N fertilizer requirement according to the German Fertilizer Ordinance (2020).

b Modifications of the N fertilizer requirement according to the German Fertilizer Ordinance (2020).

c No sensor fertilization for Maize (fertilization was carried out according to the German Fertilizer Ordinance (2020)).

**Fig. 4:**
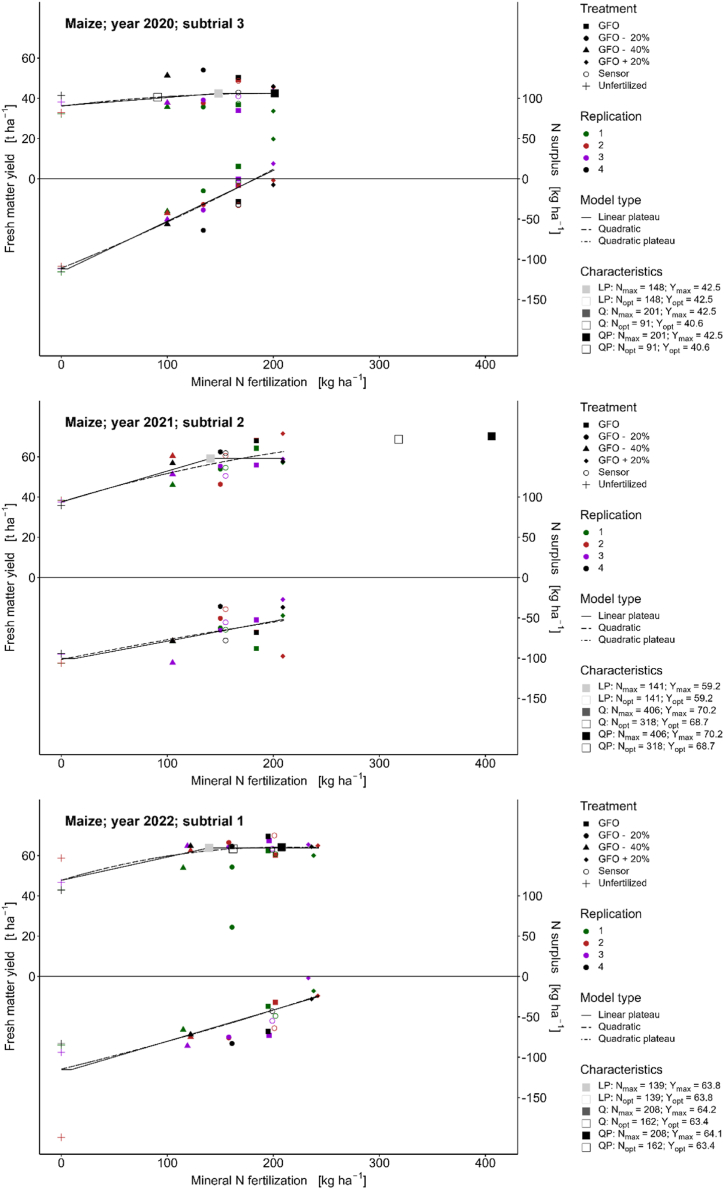
Relationships between N fertilization, yield, and nitrogen surplus for maize. Yield is given in fresh matter with a dry matter content of 32%.

The yield response curve of maize in 2020 was very flat (Fig. 4, subtrial 3). Due to the low yield response of maize, the rare case occurred that the economic optimum according to the Q and QP models (91 kg ha^−1^) was lower than the optimum according to the LP model (148 kg ha^−1^) (Table 8).

#### 3.3.2 Maize; year 2021; subtrial 2

The target yield of 55.0 t ha^−1^ was exceeded in all fertilized treatments (Table 7). In N 1 (unfertilized), a yield of 36.0 t ha^−1^ was attained. N 2 (GFO) did achieve the maximum yield with 61.7 t ha^−1^; however, this was not statistically different from N 5 (GFO +20%) (60.3 t ha^−1^), N 3 (GFO -20%) (54.5 t ha^−1^), and N 6 (Sensor) (56.2 t ha^−1^). N uptake varied between 100 kg ha^−1^ in N 1 (unfertilized) and 261 kg ha^−1^ in N 5 (GFO +20%) (highest fertilized treatment). Overall, high N use efficiency of up to 85% was reached (in N 4, GFO -40%) owing to the high yields and relatively low N fertilization. Also, in N 2 (GFO), a high N use efficiency of 83% was achieved. The lowest N use efficiency was calculated in treatment N 3 (GFO -20%) with 68% and differed statistically significantly from all remaining treatments. All treatments achieved strong negative N balances (−100 kg ha^−1^ to −52 kg ha^−1^).

Because of the high variance in yields within each treatment and still noticeable yield increases in the higher fertilizer treatments, N response functions (Fig. 4, subtrial 2) can hardly be interpreted for maize in 2021. Thus, the theoretical optimum according to the LP model was 141 kg ha^−1^ mineral N, whereas the optimum according to the Q and QP models was 318 kg ha^−1^ (Table 8).

#### 3.3.3 Maize; year 2022; subtrial 1

The target yield of 55.0 t ha^−1^ was nearly exceeded in all fertilized treatments (Table 7). The highest yield was measured in treatment N 6 (Sensor) with 53.7 t ha^−1^; however, this only differed statistically from treatment N 1 (unfertilized). Already fertilization of 120 kg ha^−1^ (GFO -40%) was sufficient to achieve maximum yield (49.7 t ha^−1^), with no statistically significant differences compared to the other fertilized treatments.

Relatively high N uptake was measured in N 1 (unfertilized) at 115 kg ha^−1^. The highest N uptake was attained in N 5 (GFO +20%) with 255 kg ha^−1^, but it showed no statistical differences with the remaining fertilized treatments. N efficiencies in this trial varied from 54% in N 3 (GFO -20%) to 68% in N 2 (GFO) and N 6 (Sensor). In all treatments, negative N balances were calculated (up to −115 kg ha^−1^).

The optimum yield in 2022 was reached with 139 kg ha^−1^ mineral N according to the LP model and 162 kg ha^−1^ according to the Q and QP models (Table 8). In 2022, higher N inputs were made in the N 2, N 5, and N 6 treatments, and fertilization according to the GFO (197 kg ha^−1^) was significantly above the economic optimum. The N surplus at the economic optimum, according to LP, amounted to −65 kg ha^−1^, and for Q and QP models, it amounted to −57 kg ha^−1^ (Fig. 4, Appendix 6).

### 3.4 Soil mineral nitrogen content and crop yield ratio

The SMN content was determined in the 0–60 cm layer at various dates, mainly in autumn (Appendix 7). For some dates (October 2021, subtrial 2, subtrial 3), low and undifferentiated SMN contents were identified. On the same date, however, higher SMN contents were found in subtrial 1 (after low-yielding winter barley), which were significantly higher in treatments with high N inputs than in those with low N inputs. In September 2022, relatively high SMN contents occurred, especially in the highest fertilized treatment N 5 (in all subtrials), which was significantly reduced by intercropping after winter barley in subtrial 3 until November 2022. SMN levels are generally related to N fertilization, N use efficiency, and N balances. In line with the low N surpluses, the SMN contents were also low.

#### 3.4.1 Crop yield ratio

The crop yield ratio of GFO to the unfertilized treatment over three years is shown in Table 9. The wheat yield felt by 71 %, the barley yield by 62 %, but the maize yield only by 28 % comparing GFO to the unfertilized N1 treatment.

**Table 8:** Yield: evaluation of the model quality of the quadratic, the quadratic-plateau, and the linear plateau functions with the indicators R^2^, RMSE, and MAE and the characteristics N_max_ ((minimum) nitrogen fertilizer amount to reach maximum yield), Y_max_ (maximum yield), N_opt_ (nitrogen fertilizer amount to obtain the maximum economic output) and Y_opt_ (yield at N_opt_). The yield is given in fresh matter with a dry matter content of 32 % for maize and 86 % for wheat and barley.

group name	model type	MAE	RMSE	R^2^	N_max_	Y_max_	N_opt_	Y_opt_
wheat 2020	LP	0.29	0.36	0.97	143.2	10.43	143.2	10.43
wheat 2020	Q	0.23	0.32	0.97	223.6	10.76	201.8	10.70
wheat 2020	QP	0.23	0.32	0.97	223.6	10.76	201.8	10.70
wheat 2021	LP	0.20	0.25	0.99	184.9	11.05	184.9	11.05
wheat 2021	Q	0.18	0.23	1.00	287.3	11.57	265.7	11.51
wheat 2021	QP	0.18	0.23	1.00	287.3	11.57	265.7	11.51
wheat 2022	LP	0.29	0.34	0.99	180.8	11.12	180.8	11.12
wheat 2022	Q	0.21	0.24	0.99	276.3	11.44	255.2	11.38
wheat 2022	QP	0.21	0.24	0.99	274.6	11.42	253.8	11.36
barley 2020	LP	0.30	0.43	0.88	74.6	9.57	74.6	9.57
barley 2020	Q	0.24	0.33	0.93	92.6	9.77	85.3	9.75
barley 2020	QP	0.31	0.43	0.88	93.7	9.56	85.6	9.54
barley 2021	LP	0.38	0.48	0.95	141.6	7.05	141.6	7.05
barley 2021	Q	0.40	0.48	0.94	195.9	7.14	176.2	7.08
barley 2021	QP	0.40	0.48	0.94	195.2	7.13	175.6	7.07
barley 2022	LP	0.39	0.49	0.96	155.7	9.19	155.7	9.19
barley 2022	Q	0.28	0.35	0.98	263.2	9.59	234.0	9.51
barley 2022	QP	0.28	0.35	0.98	263.2	9.59	234.0	9.51
maize 2020	LP	4.97	5.80	0.13	148.3	42.47	148.3	42.47
maize 2020	Q	5.00	5.80	0.13	201.4	42.51	90.8	40.59
maize 2020	QP	5.00	5.80	0.13	201.4	42.51	90.8	40.59
maize 2021	LP	4.50	5.77	0.65	140.8	59.20	140.8	59.20
maize 2021	Q	4.51	5.31	0.71	405.9	70.23	318.2	68.71
maize 2021	QP	4.51	5.31	0.71	405.9	70.23	318.2	68.71
maize 2022	LP	3.40	4.42	0.64	139.5	63.84	139.5	63.84
maize 2022	Q	3.52	4.41	0.64	208.0	64.22	162.4	63.43
maize 2022	QP	3.51	4.42	0.64	207.7	64.14	162.0	63.35
wheat mean	Q	0.18	0.23	0.99				
wheat mean	QP	0.18	0.23	0.99				
wheat mean	LP	0.22	0.27	0.98				
barley mean	Q	0.26	0.33	0.95				
barley mean	QP	0.28	0.36	0.93				
barley mean	LP	0.31	0.40	0.93				
maize mean	Q	1.39	1.66	0.49				
maize mean	QP	1.39	1.66	0.49				
maize mean	LP	1.37	1.71	0.47

**Table 9:** Crop yield ratio (%) calculated over all three years of the different crops compared to the German Fertilizer Ordinance (GFO).

Treatment Unit	Explanation	Maize [%]	Wheat [%]	Barley [%]
N 1	Unfertilized	72	29	38
N 2	GFO^a^	**100**	**100**	**100**
N 3	GFO - 20%^b^	93	94	96
N 4	GFO - 40%^b^	90	81	88
N 5	GFO + 20%^b^	100	103	100
N 6	Sensor^c^	99	100	103

a Determination of the N fertilizer requirement according to the German Fertilizer Ordinance (2020).

b Modifications of the N fertilizer requirement according to the German Fertilizer Ordinance (2020).

c Determination of the N fertilizer requirement based on multispectral sensor data and algorithm according to Maidl et al. (2011).

## 4 Discussion

### 4.1 Discussion of methods

#### 4.1.1 Site selection

The experimental results were influenced by the long-term exclusive mineral fertilization of the trial area. As neither organic fertilizers (manure, slurry, and compost) were applied nor N_2_ fixing legumes were cultivated in the past decades, a rather low N mineralization potential can be assumed. At this study site, low N mineralization from the soil N pool was detected (see yield and N uptake of treatment N 1 [unfertilized]). The relatively low N supply from the soil N pool is particularly evident for winter wheat and winter barley from the second year of the trial in treatment N 1 (unfertilized), where an N uptake of only 22–32 kg ha^−1^ and yields of 1.4–2.5 t ha^−1^ were attained (Table 5, Table 6). Previous studies at this site have measured low SMN levels and N_2_O emissions over several years during rape cultivation and after rape harvest [38], which was also attributed to low soil N turnover. Analyses of fields with long-term fertilization with slurry in southern Germany showed much higher N mineralization from the soil N pool and, therefore, relatively high yields and N uptake in unfertilized treatments [39,40]. On other sites in Germany with high N mineralization potential, N uptake of 75 kg ha^−1^ on the mean of the crop rotation was measured in long-term fertilization trials even after a 30-year trial period [41].

In the experimental station Roggenstein, the yields in the plot trials were evidently higher than the yields measured on the surrounding fields of commercial farms. The yield gaps between the experimental and field level are widely recognised [42] and can be caused by soil compaction [43], competition from adjacent tree rows or higher weed pressure [44]. These yield differences must be considered when interpreting the experimental data, as they also influence N use efficiency and N balances.

#### 4.1.2 Fertilization systems

With the revision of the German Fertilizer Application Ordinance in 2020, determining the N fertilizer requirement is mandatory in Germany [19] and is applied nationwide. The N fertilizer requirement calculated according to the GFO must not be exceeded. So far, no long-term trials (e.g., on crop rotation) have been carried out to test the effects of the GFO regulations. In this study, statements can be made on an experimental basis about which yield effects can be expected with fertilization according to GFO or according to GFO -20% in ‘polluted areas’ and which N use efficiency and N balances can be achieved.

Sensor-based N fertilization can be applied to barley and wheat [8,[45], [46], [47], [48]]); algorithms are available for these crops, which were also used in the field trial. In contrast, there are only a few sensor based fertilization algorithms for maize [49], mainly because the N fertilizer is usually applied at the time of sowing and therefore no further fine control of fertilization is possible.

In this study, a handheld multispectral sensor and algorithms for N fertilization were used. This fertilization system is based on vegetation indices and algorithms and has been verified with ground truth data [7,[50], [51], [52]]. The advantages of sensor-based systems (compared to GFO) are the consideration of plant growth during the vegetation period and the corresponding adjustment of N fertilizer quantity at later fertilization dates, but also the consideration of site-specific yield potentials [23,53,54].

Deriving the target yield is a decisive factor in all fertilization systems, even after GFO and the sensor-based system. Errors that occur here cannot be compensated for by the algorithms. In the trial, the yield potential was determined based on long-term yield measurements.

In this study, different regression models were used. While the quadratic model assumes a yield decline at N inputs above N_max_, the other models assume a yield plateau at N inputs above N_max_. The linear and quadratic plateau functions differ in the assumption of linear or quadratic yield increase. In most cases, the assumption of a linear increase leads to a lower fertilizer amount required to reach the maximum or economically optimal yield [55]. This is because the linear plateau function tends to overestimate the yield at the determined N_opt_. The calculated yield functions (Fig. 2, Fig. 3, Fig. 4) show that the regression functions differ significantly from year to year depending on the weather conditions. Thus, the optimum yield and the optimum N fertilization varies considerably from year to year and depending on the regression model chosen [56]. The yield functions for maize were much flatter (lower yield increase) than those for wheat and barley.

### 4.2 Discussion of results

#### 4.2.1 Yield effects of the fertilizer systems

From the trial results, it can be concluded that GFO is an efficient fertilization system, related to the given site and management conditions. Not only in Germany, but also in other countries, such as Denmark and France, there is also extensive debate about official fertilization systems [57].

With fertilization according to the GFO system, high yields were achieved at this trial site without risking high N surpluses. Good results have also been obtained with the GFO system in other trials in southern Germany [40].

According to the quadratic function, the economic optimum, which achieved the best fit for the parameters MAE, RMSE, and R^2^ (Table 8) with few exeptions, was not always met with GFO fertilization. For wheat, a mineral N fertilization of 19–57 kg ha^−1^ above the fertilization requirement according to GFO would have led to the economically optimal result. For barley, the optimum was almost reached in 2021, based on the GFO. For spring barley, the optimum was reached in 2020 with a mineral N application of 27 kg ha^−1^ less compared to GFO, whereas 33 kg ha^−1^ mineral N above GFO would have been necessary for winter barley in 2022. As the calculated economic optimum for maize in 2021 was substantially further from the N application rates tested in the trial, the GFO for maize could only be evaluated with the results of 2020 and 2022, where the optimum was 76 kg ha^−1^ below and 35 kg ha^−1^ above the GFO recommendation.

Apart from wheat, where the GFO fertilizer rate was always below the economic optimum, GFO fertilization did not show a systematic deviation from the economic optimal N fertilization. The GFO fertilization system is therefore well adjusted, especially as only a few parameters were required for calculation.

An increase in mineral N fertilization compared to GFO (+20% in treatment N 5) did not lead to a significant yield increase in any of the years under investigation but to higher crude protein concentration in wheat grain and higher N surpluses. A reduced fertilizer input compared to GFO (treatments N 3 and N 4) significantly reduced yields and protein levels for wheat and barley.

Using a multispectral sensor and algorithm (treatment N 6, Sensor) for N fertilization, in some cases, better results were achieved regarding yield and N use efficiency than with GFO. In 2021, for example, according to the sensor system (treatment N 6) in subtrial 1 (winter barley), no fertilization requirement was determined at the last fertilization date, which contributed to high N use efficiency (85%). Therefore, as shown in other studies, sensor-based systems can significantly increase N use efficiency [[56], [57], [58], [59]].

However, when using spectral analysis for N fertilization, optimized crop management concerning plant diseases, weeds, and a sufficient supply of other nutrients besides N to the plants is a basic requirement [59] to avoid false conclusions. This was ensured in the trial by macro- and micronutrient fertilization and plant protection adapted to specific requirements (see crop management, Appendix 1-3, exemplary shown for year 2022).

#### 4.2.2 Relationships between mineral N input and yield

The strong correlations between N fertilization and wheat and barley yield were observed in the regression functions (Fig. 2, Fig. 3, Fig. 4). Wheat responds strongly to reduced or increased N fertilization [60,61]. N is an essential nutrient and often the most yield-limiting factor for wheat production [[62], [63], [64]]. Although higher N application led to higher yields, this was not a linear relationship (see Fig. 2), and there was an economic optimum application where the additional yield increases were offset by the cost of the additional N application [65]. Protein concentration also showed a positive correlation with N fertilization.

In contrast, maize does not show these close relationships. High maize yields can also be achieved with relatively low fertilizer inputs [66]. The regression functions for maize appear different from those for wheat and barley, with a flatter slope of the curve and extremely pronounced year effects. Therefore, statements on whether fertilization according to the GFO is appropriate for maize are difficult and further trials are required.

Maize cultivation can lead to intensive N mineralization [6] and cause a negative effect on soil nitrogen and carbon pools [[67], [68], [69], [70], [71], [72]]. The level of N mineralization in maize depends on many factors, including the intensity of tillage, weather pattern, and N mineralization potential of the soil [73].

#### 4.2.3 N surplus and N use efficiency

The N surplus is an indicator of the potential loss of reactive N compounds (NH_3_, N_2_O, and NO_3_^−^) [[73], [74], [75], [76]]. The difference between grain N uptake (N output) and N fertilizer applied (N input) is the simplest method for calculating N surplus. This study did not consider other influencing factors, such as N mineralization and N immobilization [75].

In these plot trials, the fertilizer application systems achieved balanced N surpluses for wheat and barley (N 2, GFO: −15 to 40 kg ha^−1^; N 3, GFO -20%: −52 to 24 kg ha^−1^; N 6, Sensor: −41 to 42 kg ha^−1^) and thus demonstrated their suitability as a fertilizer application system to reduce N surpluses very well. In contrast, the N balances for maize were almost always negative in this study (up to −115 kg ha^−1^) and only once positive (+14 kg ha^−1^). According to previous studies, the critical N balance of 50 kg ha^−1^ may not be exceeded [76,77]. None of the crop rotations in this fertilizer trial exceeded this limit value. Even in the highest N fertilization treatment (N 5, GFO +20%), very balanced N surpluses were achieved (−1 to 24 kg ha^−1^). When fertilizing according to the economic optimum, slight to moderate negative N balances, depending on the model type (Q, QP, or LP), were calculated for this crop rotation. The Q, QP and LP models described the N-surplus within the tested fertilizer levels quite well (R^2^ ≥ 0.8), apart from maize (R^2^ = 0.41 – R^2^ = 0.87) (Appendix 6). Maize shows just a moderate increase of N surpluses with increasing N fertilization, but a high variation in N uptake.

Studies describe very different approaches to determining N use efficiency [78]; in this study, N use efficiency was determined using a difference method in relation to N 1 (unfertilized). In all study years, high N efficiencies were calculated for wheat (69–82%) and barley (57–85%). For maize, slightly lower but comparable N efficiencies were calculated for 2021 and 2022. The N efficiencies in 2020 for maize were extremely low (36–44%). The highest N efficiencies were achieved for N 6 (Sensor) for wheat (82%) and barley (85%) and N 4 (GFO -40%) for maize (85%).

#### 4.2.4 The examination of the hypotheses results in the following evaluation

Hypotheses (1) N fertilization according to GFO is close to the economic optimum.

As the quadratic N response model had the best fit, the following conclusions were drawn based on its results. For wheat, the economic optimum was always slightly to moderate above the recommendation made by the GFO. For barley, the economic optimum was met almost perfectly on average. There was a high year-to-year variation for maize; thus, a good prediction of the attainable yield and the exact N demand is difficult.

Therefore, this hypothesis is accepted for wheat and barley. No statement is possible for maize, and further experiments are required to analyze this.

Hypotheses (2) The reduction in N fertilization (GFO -20%) significantly decreases crop yields and net returns.

In the treatment GFO -20% yield drops were measured, although a high yield level was still achieved. This effect was statistically significant for wheat and barley. For maize, no differences or only minor differences were observed.

Therefore, hypothesis 2 is accepted for wheat and barley and rejected for maize.

Hypotheses (3) Sensor-based N fertilization leads to a significant yield increase compared to GFO, saves mineral N, and simultaneously reduces N balances.

Sensor-based N fertilization achieved no significant yield advantages (but also no disadvantages) compared to GFO; nevertheless, a higher NUE was achieved in each trial when the sensor-based N fertilization was applied (winter wheat 2021 and 2022, winter barley 2021 and 2022). For winter barley in 2021, very high N efficiencies were achieved since no fertilizer requirement was determined on the last fertilization date, and therefore no fertilization was carried out.

Therefore, hypothesis 3 was accepted.

Hypotheses (4) The choice of the right regression function is decisive for the determination of the optimal nitrogen amount and therefore for the evaluation of fertilization systems.

The three regression functions (linear-plateau, quadratic and quadratic plateau) used to evaluate the results of the field experiments led to different optima for nitrogen fertilization (N_opt_). The values determined for N_opt_ differ considerably (depending on the year and crop) between the linear plateau and the quadratic function (11–81 kg ha^−1^), and only marginally between the quadratic plateau and the quadratic function (1.4 kg ha^−1^). Thus, the choice of the regression function has a considerable effect on the determination of the economically optimal N fertilizer quantity (Nopt) and consequently a decisive influence on the evaluation of the fertilizer systems. When using the linear plateau function the conclusion would be that GFO determines a fertilizer requirement far above the optimum. The quadratic function and the quadratic plateau function however determine Nopt values near or even slightly above GFO. For our data and trial question the quadratic function seems to be more appropriate than the linear plateau function. This is mainly due to the fact that the linear plateau function tends to overestimate the yield at the determined N_opt_.

Hypothesis 4 is accepted.

## 5 Conclusions and outlook

Despite the discussion and criticism of the GFO, the results of the field trial show that high yields with high N use efficiency can be achieved with fertilization, according to the GFO. Regarding the site conditions (soil and climate) and management practices (arable farming) investigated in this study, it can be concluded that fertilization, according to GFO, leads to positive economic effects without environmentally hazardous N surpluses. However, it should be emphasized that the positive results obtained in this study cannot be transferred to other sites without further experimental testing. Additional field trials are necessary for these investigations, some of which have already been conducted by the authors.

To achieve high N use efficiency in agricultural practice and a sufficient reduction of the still too high N surpluses, it can be concluded based on the results achieved here.

− The derivation of realistic target yields is a basic prerequisite for correctly determining the N fertilizer requirement according to the GFO and sensor system. New digital methods can be a solution for estimating yields and deriving yield zones [79].
− Reducing N fertilization by 20% in polluted areas leads to relatively small yield losses (≈5%), but mostly in a significant reduction of protein concentration, which may increase over time. A longer trial period is required to clarify this.
− Sensor-assisted fertilization delivers very high yields with often lower N applications than GFO, which can save costs. An alternative to this is satellite-based systems, which have the advantage that no investments in expensive sensor systems are required.

In this study, the economic optimum was determined by weighing the increase in production against the additional fertilizer price to estimate the agronomically optimal amount of N fertilizer. This approach can be extended by incorporating the social costs of pollution from N fertilization to health, ecosystems, and climate to calculate the “socially optimum N rate” introduced by Blottnitz et al. [80]. Using different assumptions about prices of fertilizer and crops, N-response models, and emission factors, van Grinsven et al. [14] calculated the difference between the social optimum and farm's optimum N rate at 30–90 kg ha^−1^ in a cost-benefit calculation for nitrogen in Europe. This example shows that the derivation of the N optimum is not trivial, nor should it assessed exclusively from the perspective of crop production or the economy.

## Funding

This study was funded by the Bavarian State Ministry of Food, Agriculture, and Forestry.

## CRediT authorship contribution statement

**Martin Mittermayer:** Writing – review & editing, Writing – original draft, Visualization, Validation, Software, Methodology, Investigation, Formal analysis. **Joseph Donauer:** Writing – review & editing, Writing – original draft, Visualization, Validation, Software. **Stefan Kimmelmann:** Investigation. **Franz-Xaver Maidl:** Methodology, Conceptualization. **Kurt-Jürgen Hülsbergen:** Writing – review & editing, Writing – original draft, Supervision, Project administration, Funding acquisition, Conceptualization.

## Declaration of competing interest

The authors declare that they have no known competing financial interests or personal relationships that could have appeared to influence the work reported in this paper.
