# Supplementary material for: Effects of different nitrogen fertilization systems on crop yield and nitrogen use efficiency – Results of a field experiment in southern Germany
Source: Heliyon. 2024 Mar 14;10(7):e28065. doi: 10.1016/j.heliyon.2024.e28065 (PMC10981040; doi:10.1016/j.heliyon.2024.e28065)

**Appendix 1: Crop Management of winter wheat 2022**

| Winter wheat, 2022 | Unit |  | Comment | Date |
| --- | --- | --- | --- | --- |
| Previous crop |  | Maize |  |  |
| Wheat variety |  | RGT Reform |  |  |
| Treatment |  | **Amount** | **Fertilizer, plant protection products** |  |
| Cultivator |  |  |  | 09.10.2022 |
| Sowing (seed rate) | kg ha^-1^ | 170 |  | 11.10.2022 |
| Measuring with Tec5 Sensor | nm |  |  | 09.05.2022 |
| Measuring with Tec5 Sensor | nm |  |  | 20.05.2022 |
| N Fertilization, first application | kg ha^-1^ | see table 3 | Calcium ammonium nitrate | 15.03.2022 |
| N Fertilization, second application | kg ha^-1^ | see table 3 | Calcium ammonium nitrate | 11.05.2022 |
| N Fertilization, third application | kg ha^-1^ | see table 3 | Calcium ammonium nitrate | 08.06.2022 |
| Basic Fertilization | kg ha^-1^ | 110/300 | Potassium sulfate/Granulated plaster | 11.03.2022 |
| Basic Fertilization | kg ha^-1^ | 2.1/5.0 | Zinc sulfate | 22.03.2022 |
| Basic Fertilization | kg ha^-1^ | 5.0/0.5 | Zinc sulfate/Manganese sulfate | 13.04.2022 |
| Basic Fertilization | kg ha^-1^ | 5.0 | Zinc sulfate | 09.05.2022 |
| Application of plant protection product | l ha^-1^ | 0.6 | Broadcast | 29.10.2021 |
| Application of plant protection product | l ha^-1^ | 1/0.35 | Regulator 720/Moddus | 28.04.2022 |
| Application of plant protection product | l ha^-1^ | 1.5/3.75 | Ampera/Trimmer WG | 09.05.2022 |
| Application of plant protection product | l ha^-1^ | 1.5/0.5 | Revytrex/Comet | 17.05.2022 |
| Application of plant protection product | l ha^-1^ | 0.75 | Karate Zeon | 01.06.2022 |
| Application of plant protection product | l ha^-1^ | 1.0 | Prosaro | 08.06.2022 |
| Harvest | t ha^-1^ | 9.3^a^ |  | 25.07.2022 |

^a^ mean FM yield

| Winter barley, 2022 | Unit |  | Comment | Date |
| --- | --- | --- | --- | --- |
| Previous crop |  | Winter wheat |  |  |
| Wheat variety |  | Meridian |  |  |
| Treatment |  | **Amount** | **Fertilizer, plant protection products** |  |
| Cultivator |  |  |  | 23.09.2021 |
| Sowing (seed rate) | Grains (m^-2^) | 280 |  | 24.09.2021 |
| Measuring with Tec5 Sensor | nm |  |  | 19.04.2022 |
| Measuring with Tec5 Sensor | nm |  |  | 02.05.2022 |
| N Fertilization, first application | kg ha^-1^ | see table 3 | Calcium ammonium nitrate | 15.03.2022 |
| N Fertilization, second application | kg ha^-1^ | see table 3 | Calcium ammonium nitrate | 22.04.2022 |
| N Fertilization, third application | kg ha^-1^ | see table 3 | Calcium ammonium nitrate | 03.05.2022 |
| Basic Fertilization | kg ha^-1^ | 110/300 | Potassium sulfate/Granulated plaster | 15.03.2022 |
| Basic Fertilization | kg ha^-1^ | 2.1/5.0 | Copper sulfate/Zinc sulfate | 22.03.2022 |
| Basic Fertilization | kg ha^-1^ | 5.0 | Zinc sulfate | 13.04.2022 |
| Basic Fertilization | kg ha^-1^ | 5/0.5 | Zinc sulfate/Manganese sulfate | 28.04.2022 |
| Application of plant protection product | l ha^-1^ | 0.6/0.03 | Broadcast/Trimmer WG | 18.10.2021 |
| Application of plant protection product | l ha^-1^ | 0.6/1.5 | Moddus/Ampera | 22.04.2022 |
| Application of plant protection product | l ha^-1^ | 1.0/1.5/0.5 | Elatus Era/Folpan/Cerone | 05.05.2022 |
| Harvest | t ha^-1^ | 7.8^a^ |  | 06.07.2022 |

**Appendix 2: Crop Management of winter barley 2022**

^a^ mean FM yield

| Maize, 2022 | Unit |  | Comment | Date |
| --- | --- | --- | --- | --- |
| Previous crop |  | Winter barley |  |  |
| Wheat variety |  | P8329 |  |  |
| Treatment |  | **Amount** | **Fertilizer, plant protection products** |  |
| Cultivator |  |  |  | 14.03.2022 |
| Sowing (seed rate) | Grains (m^2^)^-1^ | 10 |  | 22.04.2022 |
| N Fertilization, first application | kg ha^-1^ | Table 3 | Calcium ammonium nitrate | 23.04.2022 |
| N Fertilization, second application | kg ha^-1^ | Table 3 | Calcium ammonium nitrate | 23.05.2022 |
| Basic Fertilization | kg ha^-1^ | 450 | Granulated plaster | 14.03.2022 |
| Basic Fertilization | kg ha^-1^ | 130 | Sulfur 77 % | 22.03.2022 |
| Basic Fertilization | kg ha^-1^ | 110/6/34 | Potassium sulfate/ Copper sulfate/ Zinc sulfate | 13.04.2022 |
| Basic Fertilization | kg ha^-1^ | 5.0 | Zinc sulfate | 03.06.2022 |
| Basic Fertilization | l ha^-1^ | 3.0 | Wuxal Multimicro | 14.06.2022 |
| Application of plant protection product | l ha^-1^ | 1.5 | Aspect | 09.05.2022 |
| Application of plant protection product | l ha^-1^ | 2.0 | Laudis | 18.05.2022 |
| Harvest | t ha^-1^ | 45.6^a^ |  | 24.08.2022 |

**Appendix 3: Crop Management of maize 2022**

^a^ mean FM yield

**Appendix 4: Fresh matter yield: comparison of the regression coefficients of the quadratic, the quadratic-plateau and the linear plateau function. Yield is in fresh matter with a dry matter content of 32% for maize and 86% for wheat and barley.**

| **group name** | **model type** | **a** | **b** | **c** |
| --- | --- | --- | --- | --- |
| wheat 2020 | Q^1^ | -0.0001147 | 0.0512923 | 5.025235 |
| wheat 2021 | Q^1^ | -0.0001155 | 0.0663569 | 2.033964 |
| wheat 2022 | Q^1^ | -0.0001190 | 0.0657278 | 2.357259 |
| barley 2020 | Q^1^ | -0.0004013 | 0.0743296 | 6.328643 |
| barley 2021 | Q^1^ | -0.0001491 | 0.0584118 | 1.415323 |
| barley 2022 | Q^1^ | -0.0001009 | 0.0531005 | 2.606682 |
| maize 2020 | Q^1^ | -0.0001571 | 0.0632552 | 36.142389 |
| maize 2021 | Q^1^ | -0.0001980 | 0.1607432 | 37.608248 |
| maize 2022 | Q^1^ | -0.0003810 | 0.1584483 | 47.747671 |
| wheat 2020 | Q^1^ | 10.7585283 | -0.0001147 | 223.553769 |
| wheat 2021 | Q^1^ | 11.5670322 | -0.0001155 | 287.326900 |
| wheat 2022 | Q^1^ | 11.4156636 | -0.0001202 | 274.611675 |
| barley 2020 | Q^1^ | 9.5636572 | -0.0003636 | 93.707202 |
| barley 2021 | Q^1^ | 7.1288057 | -0.0001499 | 195.238158 |
| barley 2022 | Q^1^ | 9.5946069 | -0.0001009 | 263.196055 |
| maize 2020 | Q^1^ | 42.5111059 | -0.0001571 | 201.365732 |
| maize 2021 | Q^1^ | 70.2294239 | -0.0001980 | 405.879384 |
| maize 2022 | Q^1^ | 64.1442893 | -0.0003797 | 207.701951 |
| wheat 2020 | LP^3^ | 10.4250000 | 0.0373126 | 143.228272 |
| wheat 2021 | LP^3^ | 11.0500000 | 0.0481427 | 184.862792 |
| wheat 2022 | LP^3^ | 11.1187500 | 0.0484687 | 180.813329 |
| barley 2020 | LP^3^ | 9.5750000 | 0.0429104 | 74.573913 |
| barley 2021 | LP^3^ | 7.0500000 | 0.0395790 | 141.600636 |
| barley 2022 | LP^3^ | 9.1875000 | 0.0424631 | 155.743895 |
| maize 2020 | LP^3^ | 42.4739583 | 0.0419495 | 148.272957 |
| maize 2021 | LP^3^ | 59.2011719 | 0.1541667 | 140.815034 |
| maize 2022 | LP^3^ | 63.8359375 | 0.1156573 | 139.457467 |

^1^ Quadratic function according to equation (6)

^2^ Quadratic plateau function according to equation (7)

^3^ Linear plateau function according to equation (8)

**Appendix 5: N-balance: comparison of the regression coefficients of the quadratic, the quadratic-plateau and the linear plateau function**

| **group name** | **model type** | **a** | **b** | **c** |
| --- | --- | --- | --- | --- |
| wheat 2020 | Q^1^ | 0.0001226 | 0.2422758 | -59.502648 |
| wheat 2021 | Q^1^ | 0.0001818 | 0.1823606 | -25.160600 |
| wheat 2022 | Q^1^ | 0.0004420 | 0.1814895 | -26.643602 |
| barley 2020 | Q^1^ | 0.0034710 | -0.1336308 | -67.547090 |
| barley 2021 | Q^1^ | 0.0022900 | -0.0464656 | -22.050650 |
| barley 2022 | Q^1^ | 0.0003660 | 0.2335008 | -31.519183 |
| maize 2020 | Q^1^ | 0.0004092 | 0.5330965 | -111.087936 |
| maize 2021 | Q^1^ | -0.0001440 | 0.2607906 | -101.751393 |
| maize 2022 | Q^1^ | 0.0002218 | 0.3207491 | -114.719231 |
| wheat 2020 | QP^2^ | -179.2109305 | 0.0001226 | -988.198454 |
| wheat 2021 | QP^2^ | -70.9032198 | 0.0001818 | -501.671982 |
| wheat 2022 | QP^2^ | -45.2746210 | 0.0004420 | -205.312314 |
| barley 2020 | QP^2^ | -66.7318213 | 0.0041254 | 30.401907 |
| barley 2021 | QP^2^ | -21.9554714 | 0.0023154 | 11.503853 |
| barley 2022 | QP^2^ | -68.7569327 | 0.0003660 | -318.951815 |
| maize 2020 | QP^2^ | -284.7063480 | 0.0004092 | -651.358008 |
| maize 2021 | QP^2^ | ^4^ | ^4^ | ^4^ |
| maize 2022 | QP^2^ | -230.6572820 | 0.0002218 | -722.920376 |
| wheat 2020 | LP^3^ | ^4^ | ^4^ | ^4^ |
| wheat 2021 | LP^3^ | -26.1249996 | 0.2297948 | 3.983927 |
| wheat 2022 | LP^3^ | -27.6749993 | 0.3522337 | 39.587855 |
| barley 2020 | LP^3^ | -68.3750001 | 0.5248358 | 53.746498 |
| barley 2021 | LP^3^ | -22.6250000 | 0.6561126 | 77.058545 |
| barley 2022 | LP^3^ | -31.8000001 | 0.3635126 | 28.468749 |
| maize 2020 | LP^3^ | -111.9999993 | 0.6304965 | 5.930653 |
| maize 2021 | LP^3^ | -100.5500048 | 0.2471347 | 12.704904 |
| maize 2022 | LP^3^ | -115.2499970 | 0.3871841 | 9.655702 |

^1^ Quadratic function according to equation (9)

^2^ Quadratic plateau function according to equation (10)

^3^ Linear plateau function according to equation (11)

^4^ For maize 2021 the QP model and for wheat 2020 the LP model coefficients could not be determined

**Appendix 6: N-balance: evaluation of the model quality of the quadratic, the quadratic-plateau and the linear plateau function with the indicators R², RMSE and MAE and resulting nitrogen surpluses when fertilizing according to N_max_ and N_opt_**

| **group name** | **model type** | **MAE** | **RMSE** | **R2** | **N surplus at N_max_** | **N surplus at N_opt_** |
| --- | --- | --- | --- | --- | --- | --- |
| wheat 2020 | LP | ^1^ | ^1^ | ^1^ | ^1^ | ^1^ |
| wheat 2020 | Q | 6.10 | 7.72 | 0.82 | 1 | -6 |
| wheat 2020 | QP | 6.10 | 7.72 | 0.82 | 1 | -6 |
| wheat 2021 | LP | 3.83 | 4.58 | 0.94 | 15 | 15 |
| wheat 2021 | Q | 3.77 | 4.46 | 0.95 | 42 | 36 |
| wheat 2021 | QP | 3.77 | 4.46 | 0.95 | 42 | 36 |
| wheat 2022 | LP | 4.90 | 5.80 | 0.96 | 22 | 22 |
| wheat 2022 | Q | 4.60 | 5.19 | 0.97 | 57 | 48 |
| wheat 2022 | QP | 4.60 | 5.19 | 0.97 | 57 | 48 |
| barley 2020 | LP | 5.33 | 7.33 | 0.80 | -57 | -57 |
| barley 2020 | Q | 4.85 | 6.55 | 0.84 | -50 | -54 |
| barley 2020 | QP | 4.88 | 6.50 | 0.84 | -50 | -54 |
| barley 2021 | LP | 9.18 | 11.80 | 0.84 | 20 | 20 |
| barley 2021 | Q | 8.43 | 11.21 | 0.85 | 57 | 41 |
| barley 2021 | QP | 8.43 | 11.21 | 0.85 | 56 | 40 |
| barley 2022 | LP | 5.78 | 7.31 | 0.93 | 14 | 14 |
| barley 2022 | Q | 5.85 | 7.23 | 0.93 | 55 | 43 |
| barley 2022 | QP | 5.85 | 7.23 | 0.93 | 55 | 43 |
| maize 2020 | LP | 11.69 | 15.66 | 0.87 | -22 | -22 |
| maize 2020 | Q | 11.76 | 15.59 | 0.87 | 13 | -59 |
| maize 2020 | QP | 11.76 | 15.59 | 0.87 | 13 | -59 |
| maize 2021 | LP | 12.55 | 17.27 | 0.45 | -69 | -69 |
| maize 2021 | Q | 12.76 | 17.26 | 0.45 | -20 | -33 |
| maize 2021 | QP | ^1^ | ^1^ | ^1^ | ^1^ | ^1^ |
| maize 2022 | LP | 21.84 | 34.16 | 0.41 | -65 | -65 |
| maize 2022 | Q | 21.60 | 34.15 | 0.41 | -38 | -57 |
| maize 2022 | QP | 21.60 | 34.15 | 0.41 | -39 | -57 |
| wheat mean | LP |  |  |  | 19^2^ | 19^2^ |
| wheat mean | Q |  |  |  | 26 | 33 |
| wheat mean | QP |  |  |  | 26 | 33 |
| barley mean | LP |  |  |  | -8 | -8 |
| barley mean | Q |  |  |  | 10 | 21 |
| barley mean | QP |  |  |  | 10 | 20 |
| maize mean | LP |  |  |  | -52 | -52 |
| maize mean | Q |  |  |  | -50 | -15 |
| maize mean | QP |  |  |  | -58^2^ | -13^2^ |
| all mean | LP |  |  |  | -14 | -14 |
| all mean | Q |  |  |  | -4 | 13 |
| all mean | QP |  |  |  | -7 | 14 |

^1^ For maize 2021 the QP model and for wheat 2020 the LP model could not be determined and therefore N_max_ and N_opt_ remained unknown.

^2^ When calculating means for maize QP and wheat LP missing values of single observations (^1^) were ignored.

**Appendix 7: Soil mineral nitrogen content, in 0-60 cm depth**

| Date |  |  | 07 October 2021 | 17 November 2021 | 14 March 2022 | 06 September 2022 | 10 November 2022 |
| --- | --- | --- | --- | --- | --- | --- | --- |
| Subtrial 1 |  | Unit | kg ha^-1^ | kg ha^-1^ | kg ha^-1^ | kg ha^-1^ | kg ha^-1^ |
|  |  |  |  |  |  |  |  |
| N 1 |  | Unfertilized | 15^a^ | 19^a^ | 19^a^ | 6^a^ | 9^a^ |
| N 2 |  | GFO^1^ | 30^c^ | 21^a^ | 23^a^ | 30^ab^ | 27^c^ |
| N 3 |  | GFO - 20%^2^ | 22^b^ | 20^a^ | 20^a^ | 15^a^ | 20^bc^ |
| N 4 |  | GFO - 40%^2^ | 23^b^ | 18^a^ | 21^a^ | 10^a^ | 16^b^ |
| N 5 |  | GFO + 20%^2^ | 38^d^ | 16^a^ | 22^a^ | 52^b^ | 44^e^ |
| N 6 |  | Sensor^3^ | 35^cd^ | 17^a^ | 20^a^ | 41^b^ | 33^d^ |
| Subtrial 2 |  | Unit | kg ha^-1^ | kg ha^-1^ | kg ha^-1^ | kg ha^-1^ | kg ha^-1^ |
|  |  |  |  |  |  |  |  |
| N 1 |  | Unfertilized | 11^a^ | 15^a^ | 17^ab^ | 21^a^ | 14^a^ |
| N 2 |  | GFO^1^ | 13^a^ | 18^a^ | 21^ab^ | 33^a^ | 13^a^ |
| N 3 |  | GFO - 20%^2^ | 11^a^ | 15^a^ | 20^ab^ | 25^a^ | 13^a^ |
| N 4 |  | GFO - 40%^2^ | 11^a^ | 19^a^ | 21^ab^ | 19^a^ | 13^a^ |
| N 5 |  | GFO + 20%^2^ | 12^a^ | 20^a^ | 24^b^ | 78^b^ | 19^b^ |
| N 6 |  | Sensor^3^ | 11^a^ | 16^a^ | 12^a^ | 32^a^ | 13^a^ |
| Subtrial 3 |  | Unit | kg ha^-1^ | kg ha^-1^ | kg ha^-1^ | kg ha^-1^ | kg ha^-1^ |
| N 1 |  | Unfertilized | 10^a^ | 13^a^ | 7^a^ | 17^a^ | 5^a^ |
| N 2 |  | GFO^1^ | 10^a^ | 12^a^ | 7^a^ | 25^a^ | 6^a^ |
| N 3 |  | GFO - 20%^2^ | 9^a^ | 13^a^ | 6^a^ | 19^a^ | 6^a^ |
| N 4 |  | GFO - 40%^2^ | 9^a^ | 13^a^ | 6^a^ | 18^a^ | 5^a^ |
| N 5 |  | GFO + 20%^2^ | 9^a^ | 14^a^ | 12^a^ | 44^b^ | 4^a^ |
| N 6 |  | Sensor^3^ | 13^a^ | 13^a^ | 10^a^ | 38^b^ | 5^a^ |

^1^ Determination of the N fertilizer demand according to German Fertilizer Ordinance (2020)

^2^ Modifications of the N fertilizer demand according to German Fertilizer Ordinance (2020)

^3^ Determination of the N fertilizer demand according to multispectral sensor data and algorithm Maidl et al. 2011

**Appendix 8: Revenue response functions for barley 2020. The functions were calculated by the corresponding yield functions (linear plateau (LP), quadratic (Q) and quadratic-plateau (QP)) multiplied with the product price (P_prod_; for barley: 170 € t^-1^ FM) less the fertilizer amount multiplied by the N-fertilizer price (P_fert_) of 1 € kg^-1^. The x-axis value of the maximum corresponds to the economic optimum fertilizer amount of the corresponding model type. The economic optimum for barley 2020 (black squares □) amounts 1553 € ha^-1^, 1572 € ha^-1^ and 1536 € ha^-1^ gross return less nitrogen costs, respectively for LP, Q and QP. It is achieved with an N fertilization of 75 kg N ha^-1^, 85 kg N ha^-1^ and 86 kg N ha^-1^, respectively for LP, Q and QP.**


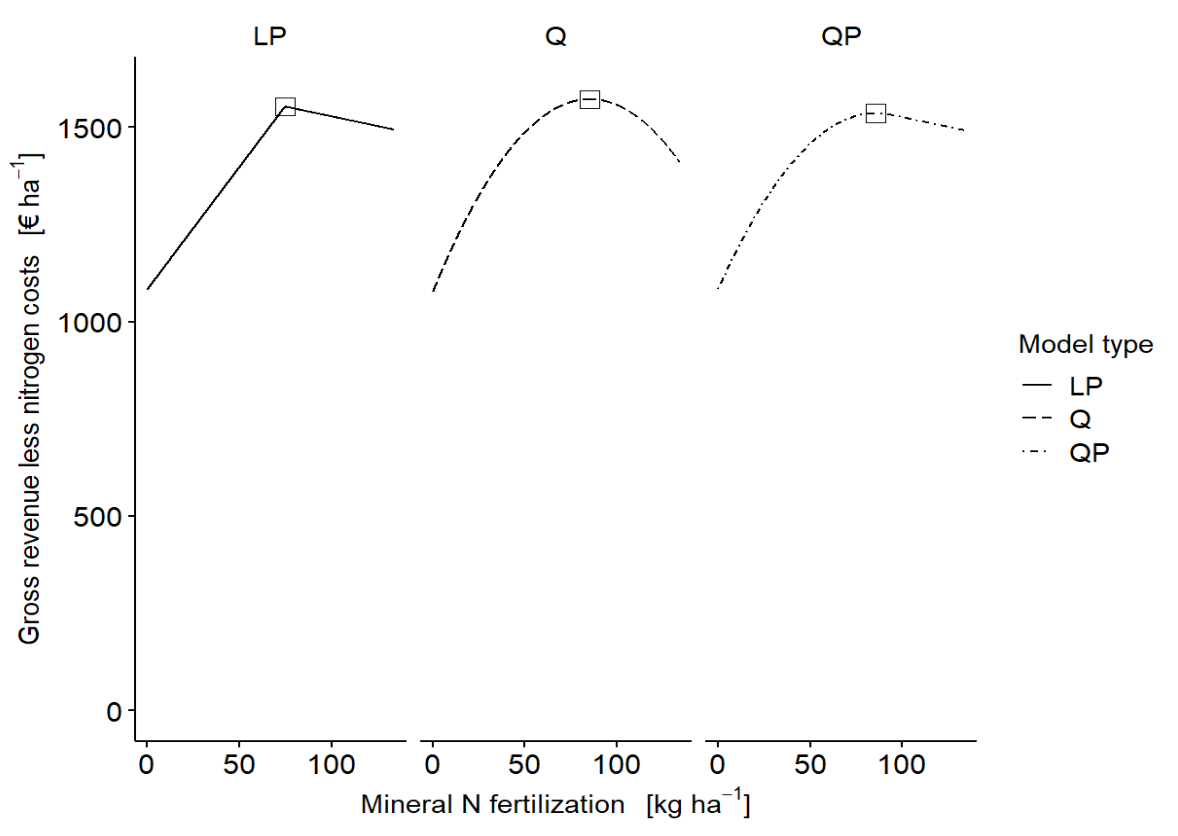

Supplement: Multimedia component 1 [file mmc1.docx]
